# Acknowledgment to Reviewers of *Cells* in 2021

**DOI:** 10.3390/cells11030477

**Published:** 2022-01-29

**Authors:** Cells Editorial Office

**Affiliations:** MDPI AG, St. Alban-Anlage 66, 4052 Basel, Switzerland

Rigorous peer-reviews are the basis of high-quality academic publishing. Due to the great efforts of our reviewers, *Cells* was able to maintain its standards for the high quality of its published papers. Thanks to the contribution of our reviewers, in 2021, the median time to first decision was 16 days and the median time to publication was 40 days. Among the reviewers who reviewed for *Cells* in 2021, the following five have been selected to receive the “*Cells* 2021 Outstanding Reviewer Awards” for the timeliness and quality of their review reports in 2021:

Bonam, Srinivasa Reddy from Université de Paris, Paris, France
Homma, Takujiro from Yamagata University, Yamagata, Japan
Hwang, In Koo from Seoul National University, Seoul, Korea
Kobeissy, Firas from University of Florida, Gainesville, FL, USA
Rebelo, Sandra from University of Aveiro, Aveiro, Portugal

The editors also express their gratitude and recognition to the following reviewers for their precious time and dedication, regardless of whether the papers they reviewed were finally published:
Abate, AndreaLiao, ChenghengAbd El-Aziz, Tarek MohamedLiao, PengAbdelalim, Essam M.Libert, ClaudeAbdel-Daim, Mohamed M.Lichtenauer, MichaelAbdolmaleki, ParvizLie, Dieter ChichungAbe, KenjiLiebau, StefanAbe, KuniyaLiedert, Astrid M.Abend, MichaelLiepins, JanisAboubakar, FrankLiepman, AaronAbraham, DietmarLifjeld, Jan T.Åbrink, MagnusLilly, MaryAbsalon, SabrinaLim, Chin YanAcosta-Alvear, DiegoLim, JaechulAcquasaliente, LauraLim, Lee WeiAdachi, YasushiLim, Sang-MooAdadi, PariseLim, Tingsen BensonAdamek-Urbańska, DobrochnaLim, WonbongAdjaye, JamesLim, YunpingAdunyah, Samuel E.Limoli, PaoloAedo Abadie, Jorge EduardoLin, BinAfanassieff, MarielleLin, Chih-HsunAfferni, ClaudiaLin, Chih-Jen (Lance)Afonso, PhilippeLin, Chih-LiAfrin, SadiaLin, ChingyuAgbarya, AbedLin, HoAguayo Paul, SebastiánLin, Jer-AnAgudo, JudithLin, LiAguilera-Tejero, EscolasticoLin, QingAgulló-Ortuño, María TeresaLin, Sheng-ChiehAhearne, MarkLin, Yung-KaiAhluwalia, BalpreetLinard, ChristineAhmad, FarazLindenberg, SvendAhmad, KhurshidLindner, Holger A.Ahmadian, M. RezaLindquist, JonathanAhn, JaegyoonLindsay, Andrew J.Aiello, SistianaLindstedt, SandraAjiro, MasahikoLiobikas, JuliusAkash, SajidLionetti, VincenzoAkihiko, OkaLipinski, PatrykAkutsu, HidenoriLipinski, PawelAlaimo, AlessandroLira, Fábio SantosAlani, EricLirussi, FrédéricAlarcón, BalbinoLisachov, Artem P.Albani, DiegoLisby, MichaelAlbert, AntonyLisowska, KatarzynaAlberti, ElenaLisowska, Katarzyna M.Alberti, PaolaLiu, AiminAlbonici, LoredanaLiu, Ai-MingAlcántara-Ortigoza, Miguel AngelLiu, ChenyingAldaz, MarceloLiu, Chia-ChiAldrich, Melissa B.Liu, ChunAldskogius, HåkanLiu, Jian-MinAlessandrini, FedericaLiu, LuningAlexander, Francis Borgio J.Liu, Michelle TianqingAlexy, TamasLiu, MingyaoAlgar, ElizabethLiu, NingAl-Ghadban, Sara I.Liu, PengdaAlhadidi, QasimLiu, Ping-YenAl-Haj-Zen, AymanLiu, Shing-HwaAli, Syed AzmalLiu, XiangshengAlibardi, LorenzoLiu, YalingAlieva, Irina BorisovnaLiu, Yen-LiangAllodi, SilvanaLiu, Yi-ChangAlmad, AkshataLiu, YieAl-Massadi, OmarLiu, Yi-WenAlmeida, David R. P.Liu, YongfengAlmeida, MarisaLiu, YonggangAlmerico, Anna MariaLiu, Yu-PengAl-Mubarak, Bashayer R.Liu, ZhenlongAlonso, JavierLjubimov, Alexander V.Alonso, Juan CarlosLlansola, MartaAlonso, Miguel A.Lleo, MarAlpers, CharlesLlobet, ArturAlpízar, Yeranddy AguiarLlorens Martin, MariaAlsaab, HashemLlorens-Bobadilla, EnricAltamura, ConcettaLloyd, R. StephenAltomonte, JenniferLo Gullo, AlbertoÁlvarez Martín, JavierLo Nigro, LucaÁlvarez-Mercado, Ana I.Lo, Chunmin C.Alvarez-Rodriguez, ManuelLo, MichaelAlvarez-Silva, MarcioLobachev, KirillAlves, C. HenriqueLobachevsky, PavelAlves, Lysangela R.Lobo, GlennAlviano, FrancescoLobo, María Val ToledoAmadoro, GiuseppinaLochhead, Jeffrey J.Amaral, Flavio A.Locker, FelixAmaral, Isabel F.Lodola, FrancescoAmasheh, SalahLoeffler, Jean-PhilippeAmbrósio, António F.Loiola, Rodrigo A.Ameele, Jelle Van DenLokstein, HeikoAmin, KawaLombardi, RaffaellaAmorim, ChristianiLombó, MartaAmpofo, EmmanuelLonardi, SaraAmthor, HelgeLonardo, AmedeoAna, Juan-GarcíaLonardo, EnzaAnderson, Peter M.Loo, ShiningAnderson, Sean A. S.Lopes, Coeli M. B.Anderson, Stacey E.Lopes, Marilene HohmuthAndón, Fernando TorresLópez Malo, DanielAndre, HelderLopez, Blanca R.Andreev, YaroslavLópez, Claudia S.Andreeva, ElenaLopez, Jose JavierAndreotti, AmyLópez, YairAngelastro, JamesLópez-Alemany, RoserAngeletti, AndreaLopez-Camarillo, CesarAngelidi, AngelikiLópez-Moya, Juan JoséAngelini, AnnalisaLopitz Otsoa, FernandoAngelini, CorradoLoppin, BenjaminAngelova, AngelinaLoppini, AlessandroAngelova, Plamena R.LoPresti, PatriziaAnghel, LarisaLorenzl, StefanAnghileri, ElenaLorenzo González, ÓscarAngrand, Pierre-OlivierLoreto, AndreaAngrisani, NinaLossius, AndreasAnsari, RaisLotz, ChristopherAntinozzi, CristinaLou, Yan-RuAntonopoulou, SmaragdiLouis, SandrineAntony, Benny S. EathakkattuLove, SethAntunes, Celia M.Low, LucieAnwar, MumtazLu, HuimingAparecida Rainho, ClaudiaLu, RichardApicella, AntonioLu, Wan-JungApolloni, SavinaLu, WenyingApostolova, NadezdaLu, Yi-ChienAppenzellar-Herzog, ChristianLu, YujiaoAragonés, JuliánLualdi, MartaAraki, ToshiyukiLubrano, ValterArata, ToshiakiLuca, GiovanniAraujo, ArturoLuci, CarmeloArber, CarolineLuciani, Giovanni BattistaAréchaga-Ocampo, ElenaLucien, FabriceArellanes-Robledo, JaimeLuddi, AliceArias, JonathanLudidi, NdikoArif, TasleemLuduena, RichardArmaiz, GuillermoLudvigsen, MajaArmant, OlivierLuganini, AnnaArmelin-Correa, Lucia MariaLuissint, Anny ClaudeArnaud, JacquelLukaszewicz-Zajac, MartaArndt, ClaudiaLukáts, ÁkosArosio, PaoloLukomska, EwaArranz, AmaiaLund, Elizabeth K.Arrebola, Francisco A.Lunder, MojcaArrigo, CiceroLüpold, StefanArroba, Ana I.Lushington, GerryArsenijevic, YvanŁuszczki, JarogniewArthur, ConnieLux, ChristopherArufe, María C.Luxton, G. W. GantAsaftei, SebastianLyck, RuthAsahara, ShunichiroLyng, FionaAsakawa, HaruhikoMa, HansongAshton, TrentMa, HongmingAsier, GonzálezMa, Suk LingAskjaer, PeterMa, Wen-JuanAspatwar, AshokMäättä, Jorma A.Aspenström, PontusMacauley, Matthew S.Assunção-Miranda, IranaiaMaccarinelli, FedericaAstigarraga, ItziarMacDiarmid, RobinAstruc, ThierryMace, Maria L.Athanasiou, Labrini V.Macek Jilkova, ZuzanaAthanassiou, PanagiotisMacEwan, DavidAtkin-Smith, GeorgiaMachado, IsidroAttanasio, ChiaraMachairiotis, NikolaosAttri, KuldeepMachin, DanielAuburger, GeorgMacIaczyk, JaroslawAudrito, ValentinaMackenzie, Louise S.Augustine, JosyMackiewicz-Milewska, MagdalenaAugustyniak, DariaMacLean, Andrew G.Auranen, AnnikaMadamsetty, Vijay SagarAurrand-Lions, MichelMadaro, LucaAust, GabrielaMadduri, SrinivasAustin, ShaneMadka, VenkateshwarAvidor-Reiss, TomerMadkour, AichaÁvila, JulioMadoui, Mohammed-AminAvila, MatiasMadrigal, IreneAvni, OrlyMadureira, Patrícia A.Avril, TonyMaeda, KazuhikoAwad, FawazMafalda, SantosAwatade, NikhilMagadum, AjitAylon, YaelMagalon, GuyAyroldi, Emira MariaMagaye, Ruth R.Azarnia Tehran, DomenicoMaggi, Leonard B.Azhar, MohamadMaggiolini, MarcelloAzimzadeh, OmidMagi, SimonaAziriova, SilviaMagin, ThomasAziz, FaisalMagnaghi, ValerioAzuma, KotaroMagnan, BrunoBabić Leko, MirjanaMagne, DavidBabiychuk, EduardMagnelli, LuciaBacete, LauraMagrassi, LorenzoBacher, UlrikeMagrì, AndreaBacker, Ronald A.Mahajan, GautamBácskay, IldikóMaia, CláudioBae, Chang-HyuMailleux, ArnaudBaer, Patrick C.Maina, FlavioBaez-Nieto, DavidMaioli, MargheritaBagalkot, TariqueMaiorano, DomenicoBaharoglu, ZeynepMaiques-Diaz, AlbaBahmad, HishamMaixent, Jean MichelBaig, MohammadMaj, MalgorzataBailis, Adam M.Majima, Hideyuki J.Bailly, MaryseMajumder, PrithaBaiocco, GiorgioMakishima, MakotoBaj, AndreinaMalagolini, NadiaBaj-krzyworzeka, MonikaMalamitsi-Puchner, AriadneBalbi, CarolinaMalapelle, UmbertoBalbi, MatildeMalashicheva, AnnaBaldanzi, GianlucaMalfa, GiuseppeBalduini, WalterMalfatti, EdoardoBalestrieri, BarbaraMalik, Anna R.Balla, AndrásMallard, BethBalla, JozsefMallela, Shamroop KumarBalogh, EnikőMalli, RolandBaloyannis, Stavros J.Mamouni, KenzaBalsinde, JesusMamun-Or-Rashid, A. N. M.Baltanás, Fernando C.Mancini, MaicolBalzamino, Bijorn OmarMancuso, SalvatriceBamburg, JamesMandl, MarkusBanerjee, HirendranathMandolesi, GeorgiaBanning, AntjeManera, UmbertoBar, Eli E.Manes, Nathan P.Barabutis, NektariosManetti, MirkoBarak, BoazManferdini, CristinaBaran, JaroslawManfredi, FrancescoBaranov, Maksim V.Manganelli, ValeriaBarbalho, Sandra MariaMangino, GiorgioBárbara, Paranhos CoelhoMani, ChinnaduraiBarbaric, IvanaMani, SridharBarbieri, FedericaManirujjaman, M.Barbosa Bessa, TheolisManivel, VivekBardag-Gorce, FawziaManna, PrasenjitBardi, GiuseppeMannion, ArleneBarghout, SamirManole, EmiliaBarichello, TatianaManzano, RaquelBarili, ValeriaManzo, EmilianoBarkan, DanielManzoor, RashidBarnstable, ColinManzotti, GloriaBaron, ByronMao, Xiao WenBaron, MartinMapelli, LisaBar-Or, DavidMaraldi, TulliaBarosova, HanaMaranesi, MargheritaBarr, Kelli L.Marasco, WayneBarraco, NadiaMarazziti, DanielaBarre, François XavierMarcello, ElenaBarreiro Iglesias, AntónMarcenaro, EmanuelaBarrera-Chimal, JonatanMarchi, SaverioBarrientos, AntonioMarcia, MarcoBarrientos, GabrielaMarconi, Guya DilettaBarrott, Jared J.Maréchal, AlexandreBar-Shavit, RachelMarei, HanyBarshtein, GregoryMargolis, LeonidBartke, AndrzejMargulis, BorisBarton, Samantha K.Maria, SirakovBartova, EvaMarian, BrigitteBarvik, IvanMarin, PhilippeBaskaran, RathinasamyMarina, Anabel IsabelBassi, Anna MariaMarini, Herbert RyanBasso, LilianMarini, Patricia EstelaBasta-Kaim, AgnieszkaMarino, JosephBastarache, JulieMarjomaki, VarpuBasu, DevrajMarkiewicz, AleksandraBathula, Chandra SekharMarkuszewski, MichałBatissoco, Ana CarlaMarone, GianniBattiato, AlfioMaroteaux, LucBattistelli, MichelaMarques, André R. A.Bauer, JohannMarques, IsabelBaufreton, JérômeMarrano, NicolaBayrak, Ömer FarukMarrelli, MassimoBeaujean, NathalieMarsch-Martinez, NayelliBeaumatin, FlorianMarselli, LorellaBecciolini, AndreaMarsh, Leigh M.Beck, Jessica A.Marshall, LindsayBecker, DoreenMarteyn, AntoineBecskei, AttilaMartin, DanielBednarek, Andrzej K.Martin, Elizabeth C.Begni, VeronicaMartín, MargaritaBehbahani, HomiraMartín-Cófreces, Noa B.Beheshti, AfshinMartínez Contreras, Rebeca DeboraBeißert, TimMartínez De Pablos, RocíoBeksac, MeralMartínez, Miguel A.Bélanger, RichardI R.Martinez, PedroBeliakova-Bethell, NadejdaMartínez-Núñez, Mario AlbertoBeling, AntjeMartínez-Rodríguez, GonzaloBelizario, José ErnestoMartínez-Sánchez, NoeliaBellavia, DanieleMartinez-Useros, JavierBellelli, RobertoMartini, AndreaBellezza, IlariaMartini, ClaudiaBello, LucaMartini, EmmanuelleBellomo, FrancescoMartins, Mariana RenovatoBelluzzi, ElisaMartín-Vasallo, PabloBelo, José AntónioMartí-Sistac, OctaviBelosludtsev, KonstantinMarullo, StefanoBel'skaya, LyudmilaMaruyama, JunkiBeltrán Frutos, EsterMaruyama, SatoshiBelvedere, RaffaellaMarx, ChristianBen Abderrahim, Mohamed AliMarzec, MichalBencúrová, ElenaMasarone, DanieleBendjennat, MouradMasola, ValentinaBenedetti, AntonioMassa, ValentinaBenesch, MatthewMassányi, PeterBenesch, Matthew G. K.Massaro, FulvioBenfaremo, DevisMassaro, GiuliaBennardo, LuigiMassart, JulieBennett, DavidMassie, AnnBennett, James P.Mastropasqua, LeonardoBentivegna, AngelaMasuzaki, RyotaBerenyiova, AndreaMáthé, CsabaBerezin, AlexanderMathieu, CoureuilBergamini, CarloMathieu, JulieBergantini, LauraMathis, Bryan J.Berge, Derk TenMathonnet, MurielBerglund, AlixMatsubara, ShigekiBerg-Sorensen, KirstineMatsubara, ShinBergstrom, KirkMatsubara, TakumaBerman, Jason N.Matsuda, YoshikoBermejo, RodrigoMatsuo, KazuyaBernabeu Aznar, MariaMatsuzaki, JunkoBernal, Juan A.Matt, StephanieBernardes, Emerson S.Mattei, VincenzoBernardi, SimonaMatthäus, ClaudiaBernardo, AntoniettaMatthews, JasonBernasconi, SergioMaturana, Carola J.Bernatik, OndrejMaugeri, AlessandroBernhardt, IngolfMaugeri, GraziaBernstein, Sanford I.Maurits, ElmerBerthelot, LaurelineMaury, WendyBertolin, GiuliaMauvezin, CarolineBertozzi, GiuseppeMauvoisin, DanielBertram, KirstieMavrogianni, Vasia S.Bertrand, FredMawhinney, ThomasBertrand, PascaleMaycotte, PaolaBertzbach, Luca D.Mayer, ArnulfBesnard, ValérieMayerhofer, ArturBesse, AndrejMazur, Antonina JoannaBesse, LenkaMazzawi, TarekBetancourt, MiguelMcAlinden, Kielan DarcyBetran, EstherMcCartney, Stephen A.Beutner, GiselaMcCaughan, GeoffBezu, LucilliaMcClements, LanaBhandoola, AvinashMcCourt, PeterBharath, Leena P.McDonough, AshleyBhardwaj, RajeshMcIntosh, J. Richard (Dick)Bhat, JaydeepMckay, TinaBhati, Kaushal KumarMcKeown, LynnBhatia, DivyaMcKimmie, CliveBhattarai, NiinaMcLean, MaryBhattarai, PrabeshMcMillin, MatthewBhawal, Ujjal KumarMeabon, James S.Bhere, DeepakMead, BenBhute, Vijesh J.Meana, ClaraBiagini, GiuseppeMeans, Robert T.Białek, AgnieszkaMears, JasonBiamonte, FlaviaMeccariello, RosariaBianchi, SerenaMechoulam, RaphaelBianchini, FrancescaMederacke, IngmarBianchini, RodolfoMedert, RebekkaBianconi, FortunatoMedica, DavideBiazzi, ElisaMedina, Kay L.Bidel, L. P. R.Medina-Rodríguez, Eva M.Bidone, Tamara CarlaMedrano, Francisco J.Bieberich, ErhardMees, MaartenBielak-Żmijewska, AnnaMegraw, Timothy L.Bieńkiewicz, AndrzejMehterov, NikolayBierhoff, HolgerMeijer, DiesBierings, Marc B.Meinhardt, MatthiasBijak, MichałMekori, YosephBikov, AndrásMeleppattu, ShimiBilban, MartinMeler, EvaBinotto, GianniMelgaço, Juliana G.Bird, DavidMellitzer, GeorgBironaité, DaivaMello, Maria Luiza S.Birtele, MarcellaMelloni, ElisabettaBirukov, KonstantinMelo, RossanaBisti, SilviaMenanteau–Ledouble, SimonBiswas, DebolinaMenchaca, Jorge LuisBizzarri, MarianoMencke, RikBjörn, Lars-OlofMéndez Bolaina, EnriqueBlagojevic Zagorac, GordanaMendez, AaronBlagojević, JelenaMendoza-Almanza, GretelBlanc, EtienneMenè, PaoloBlank, UlrichMeng, GuangxunBlanquet, VéroniqueMenis, JessicaBlas-Garciá, AnaMenna, ElisabettaBlasiak, JanuszMenon, Manoj B.Blazer-Yost, Bonnie L.Merchán, Miguel ÁngelBlazquez, Ana-BelenMerchant, JuanitaBlázquez-Castro, AlfonsoMerdes, AndreasBlondonnet, RaikoMerino, BeatrizBlumer, MichaelMerle, PhilippeBlyszczuk, PrzemyslawMeroni, GermanaBocchinfuso, GianfrancoMerrick, William C.Boehme, Karen A.Mersaoui, Sofiane Y.Boeldt, Derek S.Mert, KarakayaBoer, Cindy G.Mertens, JeromeBoersma, ArnoldMerwid-Ląd, AnnaBogaard, Harm JanMesonero, José E.Bogdanov, Ivan V.Mesquita, ThássioBogdanov, PatriciaMetere, AlessioBogie, Jeroen F. J.Methner, AxelBognár, ZsófiaMetzinger, LaurentBoguszewska, KarolinaMeyer Zu Heringdorf, DagmarBoguta, MagdalenaMeyer, IreneBoia, RaquelMeyerhans, AndreasBoisvert, François-MichelMezey, EvaBoitani, CarlaMi, LanBoix, LoretoMiano, Maria GiuseppinaBoldogh, IstvanMicha, DimitraBoldorini, RenzoMichaelsen-Preusse, KristinBollini, SvevaMicheli, FabienneBolomsky, ArnoldMichelon, WilliamBonanad, SantiagoMiddei, SilviaBonato, V. L.Miedema, Jelle R.Bonaventura, JiříMiele, Adriana E.Bonazzi, Vanessa F.Migaud, MarieBoncompagni, SimonaMigliaccio, AntimoBonfanti, LucaMigliaccio, ChristopherBongrand, PierreMihály, JózsefBönig, HalvardMika, DelphineBonner, CarolineMikoč, AndrejaBonnet, CrystelMikula, MarioBooth, Stephanie A.Milanesi, ElenaBoraschi, DianaMildner, MichaelBordin, LucianaMilena, Rondón-LagosBorek, SławomirMiliauskas, SkaidriusBorgese, NicaMilisav, IrinaBornens, MichelMiller, KarlBorriello, AdrianaMiller, Yury I.Borriello, GiovannaMiller-Kasprzak, EwaBorutinskaite, VeronikaMiloshev, GeorgeBosáková, MichaelaMilthorpe, BruceBosque, AlbertoMinafra, LuigiBoto, TamaraMinea, RaduBotta, ElenaMinelli, AlessandroBottai, DanieleMinematsu, TakeoBoucher, DaveMinguela, AlfredoBouckaert, JulieMiniero, RobertoBouitbir, JamalMinutolo, AntonellaBoulares, HamidMiranda, CatarinaBouley, RichardMiranda, ElenaBoulton, Michael E.Mirisola, MarioBouma, Gerrit JerryMishra, AartiBourgoin, SylvainMishra, Hemant K.Bourmeyster, NicolasMishra, Jay S.Bousset, LucMisiak, BłazejBoussin, FrançoisMissale, GabrieleBoussios, StergiosMistriotis, PanagiotisBoutte, Cara C.Mitchell, Cassie S.Bovolenta, PaolaMitchison, TimothyBoye, KjetilMititelu Tartau, LilianaBozgeyik, IbrahimMitola, StefaniaBozic, JoskoMitra, SumontoBrabek, JanMitsuaki, MoriyamaBraconi, DanielaMitton, KennethBraganza, AndreaMiura, ShinjiBrakenhielm, EbbaMiyamoto, IkuyaBranca, FerdinandoMiyata, JunBranca, Jacopo Junio ValerioMiyazaki, KoujiBrancaccio, AndreaMiyoshi, KeikoBrancaleoni, ValentinaMizrachi, DarioBraun, RalfMizuno, MitsuruBraun, ThorstenMizushima, NoboruBravo, Susana B.Mlynarska, AgnieszkaBrechbuhl, HeatherMo, FeiBredeloux, PierreMobasheri, AliBreitfeld, JanaMobed-Miremadi, MaryamBrenmoehl, JuliaMoby, VanessaBrennan, MeadhbhMochizuki-Kashio, MakikoBrenner, CatherineMoeinzadeh, SeyedsinaBresson-Bepoldin, LaurenceMoha Ou Maati, HamidBrestic, MarianMohamed, TamerBrindley, PaulMohammad, KhalidBrini, MarisaMohan, RamkumarBrink, PeterMohapatra, DebanandaBrinton, MargoMohibi, ShakurBrisson, LucieMohsen, MonaBrizzi, Maria FeliceMohseny, Alexander B.Brocca, StefaniaMohsin, SadiaBrockmeyer, PhillippMok, Chee KengBrodie, ChayaMoles, Jean-pierreBrodie, GrahamMolina Holgado, EduardoBroers, Jos LVMolina, Cristina E.Brogi, SimoneMollace, VincenzoBrooks, Philip JohnMollapour, MehdiBros, MatthiasMolnár, ElekBross, PeterMomekov, GeorgiBrown, DennisMoncal, Kazim K.Brown, NelsonMondal, AshisBrown, NicholasMonje-Casas, FernandoBrozovic, AnamariaMonné, MagnusBruinsma, BoteMonsalves-Alvarez, MatiasBrukman, NicolasMontalbano, MauroBrunelli, SilviaMontana, GiovannaBrunet De La Grange, PhilippeMontecucco, FabrizioBrunetti, DarioMontemurro, NicolaBrünnert, DanielaMontes, NuriaBruno, Giovanni L.Monzani, FabioBrunoni, FedericaMonzen, SatoruBruria, Ben-ZeevMoon, Eun-YiBruschini, LucaMoon, JisookBrustovetsky, NickolayMoon, MinhoBrzezińska-Błaszczyk, EwaMoon, Seung MyungBrzóska, EdytaMoraczewska, JohannaBucci, CeciliaMorais-Silva, GessyngerBüch, ThomasMorales, AlbertBuchta, ChristophMorales, JulioBuchwalow, Igor BorisowitschMorales, OlivierBucolo, ClaudioMorales, SerafinBudai-Szűcs, MáriaMorales-González, José AntonioBueno, MartaMorales-Sánchez, AbigailBuentello-Volante, BeatrizMore, ShyamBuerger, ClaudiaMoreau, ViolaineBuffo, AnnalisaMoreira, Daniel CarneiroBugarcic, AndreaMoreira, PaulaBugno-Poniewierska, MonikaMoreira, VanessaBuitrago-Molina, Laura ElisaMoreira-Filho, Carlos AlbertoBuraschi, SimoneMoreno Loshuertos, RaquelBurchell, JoyMoreno, EstefaníaBurch-Smith, TessaMoreno, SandraBurckhardt, ChristophMoreno-Andrés, DanielBurdzinska, AnnaMoretti, MartaBurelle, YanMorfoisse, FlorentBurga, Laura N.Morgan, MichaelBurger, Michael C.Morganti, ClaudiaBurgos, MiguelMorgese, Maria GraziaBurkhead, JasonMori, YasukoBurmistrz, MichalMorichi, ShinichiroBurokas, AurelijusMorimoto, TatsuyaBurster, TimoMorimura, ShigeruBusch, KarinMoriondo, AndreaBustelo, Xosé R.Moris, ArnaudButler, Daniel S. C.Moriscot, AnselmoButoi, ElenaMoriscot, Anselmo SigariBuzhdygan, Tetyana P.Morleo, ManuelaCaaveiro, JoseMoroncini, GianLucaCaballero, Raúl PérezMorozov, Alexey V.Caballo, Mar CarriónMoser, SimoneCabrera-Serrano, MacarenaMosienko, ValentinaCabrini, GiulioMosiołek, AnnaCaccamo, DanielaMotterlini, RobertoCacci, EmanueleMottet, DenisCaceres, SaraMou, HongmeiCacopardo, LudovicaMou, YongchaoCadamuro, MassimilianoMounier, CatherineCaeiro, Maria FilomenaMoustogiannis, AthanasiosCaetano, GuilhermeMravec, BorisCaffarelli, ElisaMsallam, Rasha A.Cahill, MichaelMuceniece, RutaCahill, PaulMueller, YvonneCai, KaiMukai, TomoyukiCaiazzo, MassimilianoMukherjee, RukminiCairrão, ElisaMukhtar, MahwashCalabrese, VittorioMukohda, MasashiCalabresi, LauraMuldoon, Timothy J.Calabresi, PaoloMulens-Arias, VladimirCaldwell, R. WilliamMulet, Jose M.Calhim, SaraMulherkar, ShalakaCalì, TitoMuller, Patricia A. J.Caligo, AdelaideMüller, ThomasCalina, DanielaMuniraj, NethajiCalistri, AriannaMuñoz Pinedo, CristinaCalka, JaroslawMuñoz, AnaCallaini, GiulianoMuñoz, Juan PabloCalle-Guisado, VioletaMünsterberg, AndreaCalò, AnnalisaMunteanu, Cristian V. A.Calvier, LaurentMunyard, Kylie A.Calzia, EnricoMurakami, KosakuCamaioni, AntonellaMuraro, ElenaCamargo, MarianaMurase, DaikiCambria, Maria TeresaMurayama, TakashiCameron, DonaldMuriel, PabloCammarata, FrancescoMurín, RadoslavCammarata, GiuseppeMurín, RadovanCammas, FlorenceMurooka, ThomasCamougrand, NadineMurphy, RonanCampa, Carlo CosimoMurray, RachaelCampagnolo, MartaMurthy, DivyaCampana, Luca GiovanniMusgrave, IanCampbell, Grant R.Mustelin, TomasCampbell, Lia H.Mustonen, Anne-MariCampbell, Nicole K.Mus-Veteau, IsabelleCampello, ElenaMutsaers, Steven E.Campinho, MarcoMuzza, MarinaCampo, GianlucaMyatt, LeslieCampo, Giuseppe MaurizioMyklebust, June HelenCampos-Toimil, ManuelMyllykallio, HannuCampuzano, AltheaMylonis, IliasCampuzano, OscarMyung, JihwanCanaan, StephaneNabe, TakeshiCanberk, SuleNabissi, MassimoCandiani, SimonaNadal, AlfonsCanela, Enric I.Nadal-Nicolas, FranciscoCanettieri, GianlucaNádasy, GyörgyCangiano, AlbertoNagai, MariaCanhoto, JorgeNagaoka, SatoshiCanosa, StefanoNagasaka, KazunoriCano-Sarabia, MaryNagendran, JayanCantelmo, Anna RitaNagre, NagarajaCantz, TobiasNaik, AkshataCao, HongnanNajle, Sebastián RodrigoCao, JingliNakada-Tsukui, KumikoCapanni, CristinaNakae, JunCaplan, MichaelNakagawa, TakayukiCapovilla, MariaNakagomi, TakayukiCappadocia, LaurentNakajima, HideakiCappuccilli, MariaNakajima, KeiCarafa, VincenzoNakamichi, NoritakaCarai, AndreaNakamura, TakafumiCaramujo, Maria JoséNakamura-Ishizu, AyakoCarbone, EmilioNakano, NobuhiroCarbone, MarcoNakase, HiroshiCarceller, HectorNakayama, KatsutoshiCardador-Martínez, AnabertaNakayama, MasafumiCardona, FernandoNakazawa, MitsuruCardoso, Danon ClemesNakchbandi, InaamCardoso, SusanaNalepa, IrenaCarducci, FedericaNalvarte, IvanCaretta, AntonioNam, Young-jaeCargnoni, AnnaNamba, FumihikoCariboni, AnnaNambeesan, SavithriCarina, ValeriaNamikawa, KazuhikoCarleo, AlfonsoNampoothiri, MadhavanCarlin, Cathleen R.Nan, XiaolinCarlton, JeremyNanako, KawaguchiCarman, Christopher V.Nandurkar, HarshalCarmo, AlexandreNapoli, PietroCarmody, RuaidhriNarbonne, PatrickCarmona, JuNardozza, SimonaCarnell, GeorgeNarra, SudhakarCarnevale Neto, Fausto CarnevaleNaryzhny, Stanislav N.Carnevale, RobertoNasarre, PatrickCarrascal, LiviaNashine, SonaliCarrassa, LauraNass, NorbertCarraway, Kermit L.Nassa, GiovanniCarrella, SabrinaNassir, FatihaCarreras, JoaquimNasso, GiuseppeCarrie, ChrisNastasi, ClaudiaCarrión, MarNat, Irina RoxanaCarter, Edward P.Natvig, Donald O.Carter, WayneNauli, Andromeda M.Carullo, GabrieleNava, SaraCaruntu, ConstantinNavarrete, Marcelo A.Caruso, DonatellaNavarro, EstanisCarvajal-Vergara, XoniaNavarro, Juan AntonioCarvalho, Andreia N.Naves, ThomasCarvalho, Juliana LottNawrot, BarbaraCarvalho, PedroNawrotek, PawełCarvelli, LuciaNayak, Nihar RanjanCarver, WayneNaziroglu, MustafaCasadei, NicolasNechaev, SergeiCasado-Díaz, AntonioNeelamegham, SriramCasar, BertaNeill, Stewart G.Casas, FrançoisNel, IvonneCasco, Víctor HugoNelson, Leonard J.Casini, GiovanniNémeth, AttilaCassinerio, ElenaNeuhauss, StephanCastandet, BenoîtNeumann, DetlefCastoldi, AngelaNeupane, Yub RajCastro-Munozledo, FedericoNeureiter, DanielCastro-Torres, Rubén DaríoNevler, AvinoamCatt, SallyNguyen, IsabelCattane, NadiaNguyen, PaulCausse, Sebastien. Z.Nguyen, Thi-HuongCava, ClaudiaNi, WeiCavaillé, JeromeNicholas, Ekow ThomfordCavalcanti, Marcos Fernando Xisto BragaNicita, FrancescoCavazzoni, AndreaNicoletta, BarbaniCavodeassi, FlorenciaNieland, John Dirk VestergaardCawley, NiamhNiello, MarcoCeccato-Antonini, Sandra ReginaNiemelä, Onni J.Cedeño, David L.Niemeyer, Barbara A.Čedíková, MiroslavaNiesor, Eric J.Celada, AntonioNii, TerukiCelec, PeterNiibe, KunimichiCeli, AlessandroNiikawa, HiromichiCenariu, MihaiNijakowski, KacperCenci, GiovanniNikitopoulou, IoannaCenni, VittoriaNikolaev, ViacheslavCens, ThierryNikoloudakis, NikolaosCentrone, MariangelaNikovics, KrisztinaCerasuolo, MariannaNiño-Castro, Andrea CeciliaCerdeira, Cláudio DanielNir, UriCermak, LukasNirengi, ShinsukeČerná, MarieNishida, KenseiČerný, MartinNishimune, HiroshiCervetto, ChiaraNishimura, KenCha, Hyuk-JinNissinen, Anne I.Chaiyasut, ChaiyavatNitika, NitikaChakrabarti, JayatiNobs, Samuel PhilipChakrabarti, MrinmayNocella, CristinaChakrabarti, RajarshiNofer, Jerzy-RochChakrabarty, SamitNógrádi, AntalChakraborty, ParamitaNogueira, FernandoChalas, RenataNohales, Maria A.Chan, Ben C. L.Nonoguchi, HiroshiChan, K. H. KatieNoothalapati, HemanthChan, Sunny S. K.Noren Hooten, NicoleChandra, AbhishekNoro, RintaroChang, Chuang-RungNorth, Brian J.Chang, Ling-ChuNosi, DanieleChang, Nai-JenNoureini, Sakineh KazemiChang, Nan-ShanNovopashina, DaryaChang, Sheng-NanNovotny, JiriChang, Shu-ChunNtanasis-Stathopoulos, IoannisChang, Theresa Li-YunNucera, EleonoraChang, Wei-ChaoNugraha, BramastaChang, Wei-JuNumakawa, TadahiroChang, Wen-WeiNuñez, AngelChang, Yih HsinNunzio, VicarioChao, WangNurjadi, DennisChapuis, JulienOberg, Kerby C.Charbonnier, Jean-BaptisteObeso, DavidCharbord, PierreObinata, DaisukeCharest, PascaleOcampos, Fernanda Maria MarinsCharriaut-Marlangue, ChristianeOchi, ShinichiroChateigner-Boutin, Anne-LaureOchiai, DaigoChatla, KamalakarOchiai, KazuhikoChatterjee, MadhumitaOchoa-Callejero, LauraChatterjee, ShambhabiOcker, MatthiasChaudhry, RasulO'Connell, Kevin F.Chauhan, BhareshOczkowski, MichałChavanelle, VivienOdierna, GaëtanoChávez-Calvillo, GabrielaOffermann, AnneChcialowski, AndrzejOgiwara, KatsuekiChéli, YannOgorek, RafalChen, CeshiOh, SekyungChen, Cheng-ChangO'Hara, StevenChen, Chien LunOhbayashi, NorihikoChen, Chien-ChinOhbuchi, ToyoakiChen, Chien-LiangOhe, Joo-YoungChen, Chi-ShuoOhishi, TomokazuChen, Chung-HwanOhshiro, TakahitoChen, Chung-MingOhtaka-Maruyama, ChiakiChen, Chun-JungOhya, SusumuChen, Chun-LiangOhyama, AyakoChen, Der-YuanOjha, Shreesh K.Chen, DongbaoOka, TomonoriChen, GuanOkabe, SeiichiChen, GuoxunOkada, ShigeruChen, HungwenOkajima, TetsuyaChen, JianxiongOkamoto, MarikoChen, JingshuOkazaki, YasumasaChen, JuOkbay, AysuChen, Kok SiongO'Keefe, LouiseChen, LihuaOkła, KarolinaChen, LizhenOksenych, ValentynChen, Mei-YuOláh, JuditChen, MingOlah, MartaChen, Shen-LiangOlaru, Octavian TudorelChen, Shih-HengOlchawa, Magdalena M.Chen, Shuen-EiOleari, RobertoChen, ShukunOliva, JoanChen, WeiOlivares-Reyes, Jesús AlbertoChen, XiOliveira, Karla Mychellyne CostaChen, Y. T.Oliveira, RaquelChen, Yih-FungOliver, LisaChen, YingOlivero, AntonellaChen, Yu-ChihOlmo, EttoreChen, Yung-ChiaOlsen, Abby L.Cheng, ChunchiaOlson, MichaelCheng, Hung-ChiOmarjee, LoukmanCheng, Juei-tangOmichinski, James G.Cheng, Kuang-HungOndřej, VladanCheng, Po-ChingOnizuka, SatoruCheng, Shih-LungOno, KatsushigeCheng, Shi-YuanOno, RyuichiCheng, Tsung-LinOno, YasukoCheng, Xian-wuOnoda, YusukeCheong, HeesunOnofrillo, CarmineChernoff, JonathanOnori, PaoloCherry, JonathanOntsouka, EdgarChesler, NaomiOota, KeiichiChesnokova, Vera M.Opazo, PatricioChester, Adrian H.Opiela, JolantaCheung, Wai W.Opoka, WlodzimierzCheynier, RemiOrdonez-Moran, PalomaChi, Hsiang-ChengOrellana, EstebanChi, WeiOrengo, DorcasChiarella, EmanuelaOrinska, ZaneChibon, FredericOrlandini, MaurizioChikamori, FumioOrlov, YuriChilds, Gwendolyn VaughnOrozco, LuisChimienti, GuglielminaOrtega, Angel L.Chinen, JavierOrtega-Villasante, CristinaChiramel, AbhilashOrtin-Martinez, ArturoChiu, IngmingOrtín-Martínez, ArturoChmielewski, MichalOrtis, FernandaCho, ChungheeOrtiz, AngelícaCho, HyunsooOrtiz-Masiá, DoloresCho, Myeong-CheoulOsada, Shin-IchiCho, Seong BeomOsmanagic-Myers, SelmaCho, Young-EunOsmulski, Pawel A.Choi, Dae EunOsredkar, JoškoChoi, Hae WoongOsses, NelsonChoi, Hueng-SikOstacolo, CarmineChoi, Kyung-ChulOstasov, PavelChoi, MineeO'Sullivan, JamieChoi, Youkyung H.Oswald, FranzChoi, Young WhanOtsuki, TakemiChorianopoulou, Styliani N.Ott, MariaChorostecki, UcielOttaviano, MargaretChou, Feng-ChengOtto, AngelaChoubey, DivakerOttoboni, LindaChoudhuri, SubhadipOu, C. C.Chow, PomingOuzzine, MohamedChrist, TorstenOzaki, ShujiChristian, PreußerOzretić, PetarChristiane, StehmannPacary, EmilieChristoforou, NicolasPacheco, YvesChroni, AngelikiPacherník, JiříChu, XiangpingPacia, Marta Z.Chuang, Shih-SungPacifici, RobertoChuang, YachenPacurari, MaricicaChudzinski, Ana MarisaPadler-Karavani, VeredChuffa, Luiz Gustavo De AlmeidaPagano, SabrinaChugh, Rishi ManPałasz, ArturChun, Jong TaiPalazón-García, RamiroChun, Kyung-SooPalikaras, KonstantinosChung, Byung MinPalleschi, AlessandroChung, ChaeukPallotti, FrancescoChung, ChangukPalma, Paulo J.Chung, Ching-HuPalmer, DonaldChung, SungjinPalmerini, MariaChung, Tae NyoungPalomares, OscarChutipongtanate, SomchaiPalumbi, RobertoChuva De Sousa Lopes, Susana MarinaPalyulin, VladimirChye, Mee-LenPan, Chun-HsuChyuan, I-TsuPan, JiayiCiaccio, MarcelloPan, Ming-KaiCianciosi, DanilaPanaro, Maria AntoniettaCiccarone, FabioPandey, AshutoshCicero, ArrigoPandey, SudhirCichero, ElenaPandit, AnjaliCiepiela, OlgaPanetta, DanieleCieslewicz, ArturPanizzutti, BrunaCieślińska, AnnaPannaccione, AnnaCifuentes, DanielPantopoulos, KostasCignetti, AlessandroPanzarini, ElisaCimica, VelascoPanzitt, KatrinCioce, MarioPao, Ping-ChiehCipolloni, LuigiPapa, GiovanniCippitelli, MarcoPapaccio, FedericaCirello, ValentinaPapaccio, GianpaoloCiribilli, YariPapadaki, CharaCisneros, BulmaroPapadaki, MariaCivetta, AlbertoPapadakis, Georgios E.Civita, ProsperoPapaioannou, Andriana I.Clarke, ChristopherPapait, AndreaClemens, MarkPapareddy, Ranjith K.Clerico, MarinellaPapini, AlessioCleveland, Beth M.Paplinska, MagdalenaCobley, JamesPaponov, Ivan A.Cobo-Vuilleumier, NadiaPapp, BelaCocco, LucioPapp, BernadettCoenen, Marieke J.H.Parackova, ZuzanaCohen, Ethan DavidParadowska-Gorycka, AgnieszkaCokkinos, Dennis V.Parameswaran, NeethaColanzi, AntoninoParekh, AnantColasanti, RobertoParimon, TanyalakColetti, DarioParini, AngeloColla, EmanuelaPark, DaehoCollart, Martine A.Park, Eun JeongCollas, PhilippePark, Han-ACollawn, Sherry S.Park, Jae-HyungColombini, AlessandraPark, JeonghoColosetti, PascalPark, Jong KookColunga Biancatelli, Ruben Manuel LucianoPark, Joon HaCombes, AlexPark, Ki-cheongCombettes, LaurentPark, SophieComi, CristoforoPark, Yong-JinComincini, SergioParker, Matthew F. L.Conciatori, FabianaParlatescu, IoaninaConconi, Maria TeresaParma, PietroConde, JavierParmar, MayurConfalonieri, FabriceParolini, IsabellaConfalonieri, MarcoParonetto, Maria PaolaConn, Crystal S.Parra-Damas, ArnaldoConti, PioParrilla, InmaculadaConversi, DavidParrotta, ElviraCoppola, AndreaParrozzani, RaffaeleCoraux, ChristellePascual, GloriaCorazzari, MarcoPasek, JarosławCorbí, Angel L.Passaro, FabianaCordani, MarcoPasto, AnnaCordeiro, YraimaPastorek, JaromirCordo Russo, RosalíaPatankar, Jay V.Cordo Russo, Rosalía I.Patel, Kapil D.Coria Ortega, RobertoPatel, NibeditaCormont, MireillePatel, Vaibhav B.Cornelius, DenisePatenge, NadjaCorradi, AnnaPatergnani, SimoneCorreia, SaraPatnaik, SouravCortese, BarbaraPatriarca, AndreaCortinovis, CristinaPatrussi, LauraCossarizza, AndreaPatsoukis, Nikolaos ECosta, AlexPattappa, GirishCosta, DianaPattnaik, BikashCostantini, ClaudioPaudel, Keshav RajCottrell, GraemePaudel, PradeepCoudert, JeromePaullada Salmerón, José AntonioCoulon, StéphanePaul-Visse, GesineCoulson, ElizabethPause, ArnimCourties, GabrielPautz, AndreaCoury, FabiennePauwels, LaurensCoutant, FrédéricPávek, PetrCouvineau, AlainPavlova, ElenaCowles, RobertPaw, MilenaCox, LauraPawelczyk, TadeuszCozar-Castellano, IrenePawlikowski, MarekCraiem, DamianPawlina, KlaudiaCramer, Emily R. A.Payne, AnnetteCremisi, FedericoPayre, FrançoisCrescitelli, RossellaPazgan-Simon, MonikaCriado, GabrielPeake, NicholasCrichton, Robert R.Pecqueur, ClaireCrinelli, RitaPedemonte, NicolettaCrocco, PaolinaPedone, ElisaCroce, Anna CletaPeh, Hong YongCrocè, Lory SaveriaPeinado, María ÁngelesCrocini, ClaudiaPejler, GunnarCrompton, TessaPelidou, Sygkliti-HenriettaCrook, Jeremy M.Pellet-Many, CarolineCroset, MartinePellicciari, MariaCrosio, ClaudiaPendin, DianaCross, Frederick R.Penfornis, PatriceCross, MichaelPeng, I-ChenCroteau, DeborahPenke, BotondCrow, Timothy J.Penke, LokaCuevas-Romero, EstelaPenn, RaymondCugliari, GiovanniPenning, LouisCui, HuachunPenson, PeterCui, JianzhouPentimalli, FrancescaCui, WeiPeperzak, VictorCui, WeiguoPeppelenbosch, M. P.Cukrowska, BoženaPeraçoli, Maria Terezinha SerrãoCullis, ChristopherPeraldi, PascalCulmsee, CarstenPeralta, CarmenCusick, John K.Perazzoli, GloriaCvetanovic, MarijaPerdigones, NievesCyganek, LukasPerego, CarlaCzapiewski, PiotrPereira Ferrer, ValeriaCzernicka, MałgorzataPereira, LeonelCzosnek, HenrykPereira, Patrícia AlexandraCzumaj, AleksandraPerek, BartłomiejD. Škrbić, BiljanaPeres, Lazaro Eustaquio PereiraDa Costa, Tiago JanuárioPerez Del Pulgar, SofiaDa Pozzo, EleonoraPérez, AntonioDa Silva, Henrique BorgesPérez-Campo, Flor MaríaDa Silva, JackPérez-Rubio, GloriaDa Silva, Luis Cláudio NascimentoPeriasamy, MuthuD’Agostino, MattiaPeriasamy, RameshDaha, MohamedPericàs, Juan M.Daher, João P. L.Perini, MarcosDahlin, JoakimPerišić, OgnjenDai, Dao-FuPernet, VincentDai, ZhiyuPeronnet, FrederiqueDal Prá, IlariaPerret, AlainD'Alessandria, CalogeroPerrine-Walker, FrancineD’Alessandro, MirianaPerris, RobertoD'alessio, AlessioPerucca Orfei, CarlottaDalmolin, Rodrigo Juliani SiqueiraPerucci, Luiza O.D'Alò, FrancescoPesovic, JovanDamiani, GiovanniPestov, DimitriDamiano, FabrizioPeterson, Eliza J. R.D’Amico, EmanuelePethő, GáborDandawate, PrasadPetit, Patrice X.D'Andrea, VitoPetkov, StoyanDang, Tuyen T.Petrak, JiriDanser, JanPetrenko, VolodymyrD'Antongiovanni, VanessaPettit, KristenDar, Mohd SaleemPeyronnet, RémiDarabi, RadbodPfitzner, EmanuelDarche, FabricePhan, Thi Tuong VyDasanna, Anil K.Piancone, FedericaDasgupta, BiplabPiao, WenjiDash, SabyasachiPiastowska-Ciesielska, AgnieszkaDatta, SandipanPicart, ThiebaudDaub, SteffenPiccionello, Antonio PalumboDaubon, ThomasPiccioni, AndreaDavid Cordonnier, Marie HélènePiccirillo, RosannaDavid, Laurent I.B.Piccoli, MarcoDavid, RobertPickering, RaeleneDavis, Benjamin MichaelPicketts, David J.Davolos, DomenicoPicone, PasqualeDawid, CorinnaPieretti, StefanoDay, Andrew S.Pierno, SabataDay, Regina M.Pieroni, MaurizioDayem, Ahmed AbdalPietras, AlexanderDe Almeida, Roberto FarinaPietras, EricDe Araujo, Heloisa Sobreiro SelistrePievani, AliceDe Assis, Leonardo V. M.Piffoux, MaxDe Barrios, OriolPileczki, ValentinaDe Bellis, LuigiPileri, AlessandroDe Benedetti, StefanoPilette, CharlesDe Bortoli, MarziaPiliponsky, AdrianDe Brevern, Alexandre G.Pimienta, Rodney LacretDe Carvalho, Litia AlvesPinart, ElisabethDe Castro, GabrielaPineda, José R.De Ceballos, Maria L.Pinheiro, Paulo S.De Ciuceis, CarolinaPink, DanielDe Filippis, VincenzoPinós, TomásDe Francesco, Ernestina MariannaPinto, José RenatoDe Francesco, FrancescoPinto, LuisaDe Francisco, PatriciaPinto, Maria JoanaDe Freitas Fraga, Hugo PachecoPiperi, ChristinaDe Groot, ArjanPirkmajer, SergejDe Jonghe, KrisPisarev, VladimirDe Juan Romero, M. Del CaminoPisciotta, AlessandraDe La Rosa, JorgePiscitelli, FabianaDe La Serna, Ivana L.Pissarek, MargitDe Lima e Martins Lara, NathaliaPistello, MauroDe Lourdes Pereira, MariaPistis, MarcoDe Luca, AngelaPistocchi, Anna SilviaDe Luca, PaolaPittalà, ValeriaDe Maagd, RuudPiubelli, ChiaraDe Martin, SaraPiva, FrancescoDe Masi, LuigiPizzato, MassimoDe Noronha, LuciaPlatt, Jeffrey L.De Oliva, Samara UrbanPlatta, Harald W.De Pace, RaffaellaPlayford, Martin P.De Re, VallìPlaza-Zabala, AinhoaDe Rivero Vaccari, Juan PabloPleniceanu, OrenDe Rosa, FrancescoPlotegher, NicolettaDe Rossi, PierrePlotnikov, EgorDe Ruijter-Villani, MartaPlotton, IngridDe Servi, StefanoPluta, RyszardDe Silva, PushpamaliPo, AgneseDe Sire, AlessandroPocheć, EwaDe Stefani, DiegoPoillet-Perez, LauraDe Vitis, ClaudiaPóka, RóbertDe Vito, DanilaPol, JonathanDe Vos, JohnPolacheck, WilliamDe Vries, Martine CharlottePolakowski, Nicholas J.Dean, CharlottePolańczyk, AndrzejDechat, ThomasPolge, CécileDed, LukasPolverino De Laureto, PatriziaDeftu, Alexandru-FlorianPoma, AdolfoDegano, Irene R.Poma, AnnaDeineko, Elena VictorovnaPomierny-Chamioło, LucynaDel Bene, FilippoPomorski, PawełDel Fresno Sánchez, CarlosPoncet, DelphineDel Grosso, AmbraPongratz, GeorgDel Pozo, VictoriaPonte, NunoDeLeon-Pennell, Kristine Y.Pontes, BrunoDelgado-Vélez, ManuelPoon, IvanDelhanty, PatricPoór, PéterDelitala, MarcelloPop, LaurentiuDella Pepa, GiuseppePopa-Wagner, AurelDell'Amore, AndreaPopescu, Bogdan O.Delle Monache, SimonaPopova, BlagovestaDemiot, ClairePopper, Helmut H.Demuro, AngeloPorat-Shliom, NatalieDemyanenko, Svetlana A.Porciatti, VittorioDenisov, EvgenyPors, KlausDenolly, SolènePorta, HelenaDeppermann, CarstenPorteu, FrançoiseDeres, LaszloPorzio, ElenaDerler, IsabellaPost, MarkDerouiche, AminPotaczek, Daniel P.Derudder, EmmanuelPotes, YaizaDes Rieux, AnnePotokar, MajaDeschenes, MichaelPotrony, MiriamDetsika, Maria G.Pouget, PierreDeutsch, MelaniePoulios, StylianosDev, ApurbaPouliot, RoxaneDev, SomPoulsen, KyleDevers, EmanuelPoupot, RemyDevesa, JesúsPoyner, DavidDeVito, NicholasPozzo, FedericoDevost, DominicPradet-Balade, BerengereDey, AdwitiaPradhan, DineshDey, KamolPradhan, SriharsaDey, NandiniPrakash, HridayeshDhar Dwivedi, Shailendra KumarPrakash, NilimaDhasmana, AnupamPramanik, ArindamDhungel, BijayPrantl, LukasDi Bernardo, GiovanniPrasad, KasavajhalaDi Certo, Maria GraziaPrêle, Cecilia M.Di Cesare Mannelli, LorenzoPrestileo, TullioDi Donato, MarziaPreta, GiulioDi Felice, ValentinaPriante, GiovannaDi Fonzo, AlessioPriel, AviDi Franco, SimonePrieto, Rafael MariaDi Giacomo, ClaudiaPrigent, ClaudeDi Giammartino, Dafne CampigliPrimožič, InesDi Gioacchino, MarioPritchard, MicheleDi Girolamo, NickPritesh, JainDi Meglio, FrancaPrivette Vinnedge, Lisa PrivetteDi Paola, RosannaProchowska, Sylwiadi Patti Maria Carmela, BonaccorsiProcopio, Francesco AndreaDi Pietro, PaolaProdromou, ChrisostomosDi Pietro, RobertaProescholdt, MartinDi Ruscio, AnnalisaProkop, Jeremy W.Diamanti, LucaProndzynski, MaksymilianDíaz-Payno, PedroProsperi, JeniferDickinson, RobertProtchenko, OlgaDidangelos, TriantafyllosProvost, PatrickDiederich, SandraPruijm, Menno T. C.Diederichsen, Louise PyndtPrzybyło, MałgorzataDietz, Robert M.Pucci, FabrizioDiez Del Corral, RuthPucino, ValentinaDihazi, HassanPuglisi, RossellaDimakopoulos, YannisPujia, ArturoDimitriadis, GeorgePullen, NicholasDimitroff, Charles J.Pulliero, AlessandraDimopoulos, Meletios-AthanasiosPuniya, Bhanwar LalDinescu, SorinaPurchase, CraigDing, BaojinPusch, StefanDing, Ling-WenPüschel, Gerhard P.Dioguardi, Francesco S.Pushchina, EvgeniyaDiwan, AbhinavPustylnyak, VladimirDix, RichardPuthenparampil, MarcoDiz-Chaves, YolandaPuukila, StephanieDjerada, ZoubirQasim, MuhammadDjonov, ValentinQi, XiaoyangDmitrieva, Renata I.Qi, YueDmuchowska, DianaQian, YanrongDo Carmo, SoniaQu, LiliDo, Minh TruongQu, NingDoberentz, ElkeQuaglino, DanielaDobolyi, ArpádQuax, PaulDobrowolski, PiotrQuerol Audí, JordiDobrzynski, HalinaQuesada, AntonioDoetsch, Paul WilliamQuintanilla, Rodrigo A.Dohgu, ShinyaRaasakka, ArneDokudovskaya, SvetlanaRabelink, TonDolivo, DavidRacaud-Sultan, ClaireDolzani, PaoloRachek, Lyudmila I.Domenici, FabioRacioppi, LuigiDomeniconi, Raquel FantinRaczkowska, JoannaDomingos, Pedro M.Radælli, EnricoDominguez-Vías, GermanRademaker, MariusDomon, HisanoriRadenkovic, MiroslavDonahue, Seth W.Radinger, MadeleineDonati, GiacomoRadisavljevic, ZivDondossola, DanieleRadosavljević, TatjanaDoria, RossellaRadu, Beatrice MihaelaDormond, OlivierRageul, JulieD'Oronzo, StellaRaggi, PaoloDos Santos, Reinaldo SousaRaghav, Pawan KumarDoss, Michael XavierRaghunath, MichaelDosset, MagalieRagni, EnricoDoulias, Paschalis-ThomasRagusa, Michael J.Doustkhah Heragh, EsmaeilRahaghi, Franck FarzadDovat, SinisaRahman, M. AzizurDoye, ValérieRahman, Masmudur M.Dragoni, SilviaRai, VikrantDrenjančević, InesRaimondi, LaviniaDreux, MarleneRaimondo, DiegoDriesen, Ronald B.Rajagopal, SudarshanDrlica, KarlRajamohan, ArunDrouet, EmmanuelRajendran, PeramaiyanDrozd-Sokołowska, JoannaRajendran, Ramya LakshmiDrucker, MartinRajendren, SubaDrukarch, BenjaminRakic, PaskoDrulis-Fajdasz, DominikaRakonczay, ZoltánDu, HengRalvenius, WilliamDu, ShaojunRamachandra, ChrishanDu, Xiao-JunRamadori, GiulianoDu, XimingRamalho-Santos, JoãoDuarri, AnnaRamírez Sebastián, Ana I.Duarte, Marcio S.Ramos, Ana PaulaDubinin, MikhailRamos, CarlosDubovy, PetrRamprasath, TharmarajanDubrova, YuriRamsden, DavidDubský, MichalRamsköld, DanielDubuquoy, LaurentRanganna, KasturiDucamp, SarahRangel, Luciana PereiraDucret, ThomasRanieri, GirolamoDudzińska, EwaRanjit, SabinaDuerr, ClaudiaRanzato, EliaDuguez, StephanieRao, MangalaDukhinova, Marina S.Rapino, CinziaDumas, Sébastien J.Rappeneau, VirginieDumbauld, ChelsaeRasin, Mladen RokoDuncan, Greg J.Rašin, Mladen RokoDuncan, Raymond ScottRasmussen, LeneDuncan, Steven R.Rasola, AndreaDunlop, ElaineRassow, JoachimDuntas, LeonidasRastegar, MojganDupont, GenevièveRasul, SazanDurand, BénédicteRatajczak, Mariusz ZDurek, ThomasRathinasabapathy, AnandharajanD'Uscio, Livius V.Rato, LuísDutt, Taru S.Ratti, StefanoDutta, PranabanandaRatushnyy, AndreyDutta, SumanRaut, Pawan KumarDuvigneau, J. CatharinaRavaioli, FrancescoDux, MáriaRavasio, AndreaDvorak, ZdenekRavens, SarinaDye, Danielle E.Ravens, UrsulaDyer, ArthurRayburn, Edward B.Dymova, MayaReal, Luis MiguelDziedziech, AlexisRébé, CédricDziegiel, PiotrRecsan, ZsuzsannaDziewiȩcka, MartaReddy, SangeethaEar, JasonRedell, Michele S.Eaton, DouglasReggiani, CarloEbeed, HebaReggio, AlessioEbstein, FrédéricRehman, Attiq UrEckhardt, MatthiasReichardt, Holger M.Eckhart, LeopoldReiff, TobiasEder, Iris E.Reinach, Peter S.Eeckhoute, JérômeReindl, WolfgangEfrat, ShimonReineke, LucasEftekharpour, EftekharReiner, OrlyEgan, Colin GerardReiss, Allison B.Ehinger, Johannes K.Reitman, MarcEhlting, ChristianRelat, JoanaEhrlich, Aliza TobyRelvas, JoaoEid, NabilRemmerswaal, Ester B. M.Eide, DavidRenaut, JennyEiró, NoemíRepetto, Marisa GabrielaEisel, Ulrich L. M.Resch, UlrikeEixarch, HerenaReshkin, StephanEjaz, AsimReshkin, Stephan JoelEl Alaoui, HichamReuken, Philipp A.El-Aarag, Bishoy Y. A.Reutov, ValentinElchaninov, AndreyReuveni, MosheEldridge, SandyRevechon, GwladysEleftheriou, Eleftherios P.Revuri, VishnuElena, AdinolfiReyes, José LuisEl-Esawi, Mohamed A.Reynaert, Niki L.El-Far, Ali H.Režen, TadejaEliseeva, IrinaRezoagli, EmanueleEljaafari, AssiaRezus, ElenaEljaszewicz, AndrzejRhee, KunsooElKamhaw, AhmedRibas, VicenteEl-Khamisy, SherifRibeiro, LuísEllepola, KassapaRibeyre, CyrilEllis, RonaldRibitsch, IrisEl-Mochtar, ChoaaRicci, ErikaElmonem, MohamedRicciotti, EmanuelaElroy-Stein, OrnaRichard, BodnarElsayed, MansourRichards, AllisonElsherbiny, NehalRichards, Dylan JackEl-Shishtawy, MamdouhRichter, Günther H. S.Elvire, Pons-TostivintRichter, VladimirEmery, R. J. NeilRicklefs, Franz LennardEmmerson, ElaineRidrigo, AnaEmons, GünterRiederer, PeterEngeland, KurtRiera, MartaEngström, YlvaRigoni, MichelaEnguita, Francisco J.Rijavec, MatijaEoli, MaricaRiku, YuichiEpp, JonathanRim, YeriEppard, ElisabethRinnerthaler, MarkErickson, DavidRiobo, NataliaEricson, Marna E.Riou, Laurent M.Erjavec, Gordana NedicRitz, UlrikeEscolà-Gil, Joan CarlesRius-Pérez, SergioEscors, DavidRiuzzi, FrancescaEscribese, María M.Riva, NiloEskelinen, Eeva-LiisaRivera-Toledo, EvelynEsmaili, MansooreRivero, FranciscoEsnaut, StephaneRivero-Müller, AdolfoEspada, JesúsRivoltini, LiciaEspana, EdgarRizzarelli, EnricoEspel, EnricRizzi, RobertoEspíndola, OtávioRizzo, AlessandroEspinosa, LluisRo, HyunjuEsposito, GiuseppeRobak, TadeuszEsser, NathalieRobert, DavidEsteban, MaríaRobert, Philippe A.Esteban, RaquelRoberto, MichelaEsteban, VanesaRobertson, AvrilEsteves, Ana RaquelRobertson, ShellyEstus, StevenRobin, François B.Eto, YoshikastuRobinson, MathewEufemi, MargheritaRocha, Emily M.Eugenin, EliseoRochaix, Jean-davidEun, Hyuk SooRoche, SandraÉva, CsabaRochfort, KeithÉva, MikóRöder, StefanEvani, Shankar JaikishanRoderburg, ChristophF. G. Dias, FernandaRodrigo, LuisFabczak, HannaRodrigue, AgnèsFabi, João PauloRodrigues, Alice C.Fabian, ZsoltRodrigues, Fernando José SantosFabiana Fernandes, BressanRodrigues, Pedro MiguelFacchiano, AntonioRodrigues, TeresaFacchin, FedericaRodriguez, Anne-marieFaciotti, FedericaRodriguez, RafaelFafián-Labora, Juan A.Rodriguez, RaphaëlFaget, JulienRodríguez, Ricardo ReyesFagone, PaoloRodríguez, Víctor ManuelFahad, ShahRodriguez-Blanco, JezabelFahrner, MarcRodriguez-dorantes, MauricioFailla, Cristina M.Rodriguez-Fernandez, Jose LuisFais, StefanoRodríguez-Kessler, MargaritaFaissner, AndreasRodríguez-Pallares, JannetteFajkus, JiříRodríguez-Vita, JuanFalasca, LauraRodríguez-Yoldi, MaríajesúsFalkmer, Ursula Gerda IngeRoduit, RaphaelFall, MamadouRoe, Judith L.Fang, Evandro FeiRoelen, BernardFang, QingmingRoger, LaurélineFang, Su-ChiungRogers, Ian M.Fang, Yu-WeiRog-Zielinska, Eva AlicjaFantini, DamianoRoh, Gu SeobFarcasanu, Ileana C.Roh, SanghoFaris, Pawan SirwanRohde, DavidFarkaš, VladimírRohlena, JakubFarmer, DianaRojiani, Mumtaz V.Farnsworth, Nikki L.Rojo, EnriqueFaron-Górecka, AgataRojo, Maria AngelesFarooqi, Ammad AhmadRoma, CristinFasciana, TeresaRomanel, AlessandroFasolato, CristinaRomanelli, Maria GraziaFatkhudinov, Timur Kh.Romani, PatriziaFaustini, GaiaRomaniello, DonatellaFavaloro, Emmanuel J.Romanin, ChristophFavi, EvaldoRomanini, AntonellaFaviana, PinucciaRomano, ArturoFavier, JulienRomano, CorradoFehrenbach, DanielRomano, Giovanni LucaFeith, DavidRomano, MarcoFejtova, AnnaRomano, NiclaFekete, TündeRomanyuk, NataliyaFelcht, MoritzRomei, CristinaFeldo, MarcinRomeo, Maria AneleFeliciani, ClaudioRomero, Marta R.Félix, Luís M.Romero-López, CristinaFeltham, RebeccaRomero-Lopez, MonicaFendt, MarkusRona, RobertoFeng, BingjianRonca, RobertoFeng, Sheng-WeiRondeau, PhilippeFeola, AndrewRosado, Iván ValleFereira Garrudo, Fábio FilipeRosado, JuanFerguson, StephenRosas-Vargas, HaydeéFerlazzo, NadiaRosati, ElisaFernandes, HugoRosati, JessicaFernández Galilea, MartaRoşca, Ana MariaFernández Ponce, Cecilia MatildeRoscini, LucaFernandez, EduardoRose, Alan B.Fernandez, Marina O.Rosenberg, MaiFernández, Sara Rosalía MorcuendeRosenspire, A. J.Fernández, Vanesa MartínRosenthal, CynthiaFernández-Albarral, José A.Rosenthal, Dean S.Fernández-Carrión, RebecaRosetti, FlorenciaFernández-Martínez, EduardoRosi, AntonellaFernández-Sánchez, LauraRosi, SusannaFernández-Solà, JoaquimRosina, MarcoFernandez-Twinn, DeniseRossa, Carlos, Jr.Ferree, PatrickRossi, ElisabettaFerreira Cerca, SebastienRossi, GianpaoloFerreira, FernandoRossi, RacheleFerreira, JorgeRossignol, JulienFerreira, RodrigoRosso, ChiaraFerreira, VivianaRossowska, JoannaFerreira-da-Silva, FranciscoRota, PaolaFerrucci, MichelaRotblat, BarakFessler, JohannesRoth, StevenFesus, LaszloRothhammer, VeitFiedorowicz, EwaRotstein, Benjamin H.Figueiredo, BonaldRotti, PavanaFilatov, AlexanderRouleau, MatthieuFilek, MariaRoumenina, LubkaFilep, János G.Roux, KyleFiligheddu, NicolettaRovatsos, MichailFilipek, AgnieszkaRoy, MiltonFilipek, AnnaRozalski, MarcinFilipič, BratkoRozanowska, MalgorzataFilipovic, NatalijaRozentryt, PiotrFilippou, Panagiota S.Rozman, PrimozFilosa, JessicaRozzo, CarlaFindlay, Geoffrey D.Ruaro, BarbaraFinelli, CarmineRubelj, IvicaFinkenzeller, GünterRubilotta, EmanueleFinocchiaro, GaetanoRubino, Federico MariaFinsterer, JosefRugonyi, SandraFior, RitaRump, KatharinaFiorani, MaraRund, DeborahFiorotto, RominaRurali, EricaFirestein, Bonnie L.Rusciano, DarioFirestein, RonRusciano, Maria RosariaFischer, AndreasRusek, MartaFischer, Michael B.Rusin, MarekFischer, UlrikeRusmini, PaolaFisher, HeidiRussell, OliverFiszer, AgnieszkaRusso, Matteo A.Fitzhugh, KirkRusso, TommasoFlagg, ThomasRuzafa, NoeliaFleming, AlastairRuzov, AlexeyFleming, CassandraRuzza, PaoloFletcher, Nicola F.Ryazansky, SergeiFlockerzi, VeitRybska, MartaFlorán, BenjamínRybtsov, StanislavFlorean, CristinaRyczek, NataliaFlores, AnaRyffel, BernhardFogacci, FedericaRyskalin, LarisaFolco, GiancarloRzepka, ZuzannaFollenzi, AntoniaRzymowska, JolantaFonseca-Alves, Carlos EduardoSabater, LidiaForini, Francesca S.Sabbatinelli, JacopoForleo, Giovanni B.Sabbatini, Maria EugeniaForma, AlicjaSabol, MajaFornaguera, CristinaSacco, ElenaFornasaro, StefanoSaccone, SalvatoreForni, MonicaSacedón, RosaForoncewicz, BartoszSachadyn, PawełForrest, DouglasSachinidis, AgapiosForstmeier, WolfgangSack, UlrichFort, PhilippeSadagurski, MariannaForte, MaurizioSadasivam, MohanrajFortschegger, KlausSadeghi, SaeedFortunel, NicolasSadhasivam, BalajiFoschino-Barbaro, Maria PiaSadhukhan, PritamFossarello, MaurizioSadoul, KarinFossati, SilviaSaeki, KumikoFowkes, RobSaeki, ToshiakiFragoso, RitaSaez, FabriceFraineau, SylvainSaéz, Juan CarlosFrancavilla, ChiaraSáfrány, GézaFrancés, RubénSagdullaev, Botir T.Francis, HeatherSaglio, GiuseppeFranco, AntoniettaSah, Bert-RamFranco, OmarSah, NirnathFranco, PierfrancescoSaha, ShekharFranco, SantosSaha, Sushanta KumarFrancs-Small, Catherine Colas DesSahgal, PranshuFrank, SamuelSaid, Elias A.Frankel, AdamSaint-Pol, JulienFransen, Marieke F.Sainz, BrunoFranzin, RossanaSaisho, YoshifumiFrasca, LoredanaSaito, TakashiFraschini, RobertaSakai, KenFrassanito, AntonellaSakai, ShinjiFrati, AlessandroSakamoto, AtsushiFreen-van Heeren, JulianSakamoto, YuzuruFreitas, Vanessa MoraisSaksena, NitinFreude, KristineSakwe, AmosFriant, SylvieSala, ClaudiaFrick, LucianaSala, MariaelvinaFridrichova, IvanaSalamon, DominikaFriis-Hansen, LennartSalehi, S. AlbertFrisoni, TommasoSalinas, EvaFrontiñán-Rubio, JavierSalman, MootazFrontini, AndreaSalomé Pires, AnaFu, MinguiSalton, FrancescoFuchs, MarcSalvadori, SusannaFuentealba, JorgeSalvatorelli, EmanuelaFujii, HidekiSalvetti, AnnaFujii, KazuyasuSamaddar, MadhujaFujii, MasayukiSamaja, MicheleFujimura, AtsushiSamarzija, IvanaFujishima, HiroshiSamasca, GabrielFujita, AkikazuSambri, AndreaFujitani, MasashiŠamec, DunjaFukagawa, TatsuoSamec, MarekFukasawa, TakemichiSamidurai, ArunFukazawa, TakuyaSampaio-Marques, BelémFukushima, AtsushiSampetrean, OlteaFulci, ValerioSamuchiwal, Sachin K.Fulgenzi, GianlucaSánchez, AránzazuFunaki, MakotoSánchez-Mendoza, AliciaFuoco, ClaudiaSánchez-Pernaute, RosarioFuruse, JunjiSandoval-Acuña, CristiánFusar-Poli, LauraSandrim, Valéria CristinaFuster, DanielSaneyasu, TakaokiFutrega, KathrynSanfeliu, CoralGabrielli, ArmandoSang, YongmingGabrielli, BrianSangadala, SreedharaGabryelska, MartaSangiolo, DarioGad, AhmedSankaran, Vijay G.Gad, Annica K. B.Santaeufemia, SergioGaddam, Ravinder ReddySantamaria-Martínez, AlbertGaffke, LidiaSantarelli, AndreaGage, MatthewSanthoshkumar, PutturGai, MartaSantinon, GiuliaGaidano, GianlucaSantos, Joana M. O.Gaillard, AfsanehSantos, MarianaGaillard, HélèneSantulli, GaetanoGaillochet, ChristopheSanz, CarmenGalbiati, MariaritaSanz, PascualGalbiati, SilviaSapienza, DanielaGalderisi, UmbertoSaraiva, Miguel MascarenhasGalešić, KrešimirSarantseva, SvetlanaGalietta, LuisSardanelli, Anna MariaGaligniana, Mario D.Saretzki, GabrieleGallardo, M. EstherSarkar, ArijitaGallardo, NildaSarkar, GobindaGallego, MonicaSarkar, SusobhanGallego, Óscar S.Sarma, PranjalGallo, GaetanoSarnowska, ElzbietaGallorini, MarialuciaSastre, LeandroGaloian, KarinaSato, PriscilaGaluska, Sebastian P.Sato, TakeshiGaman, Mihnea-AlexandruSatou, RyousukeGamarra, LionelŠatović, EvaGambaryan, StepanSaucedo-García, MarianaGamboa-deBuen, AliciaSauma, DanielaGamez, StephanieSavary, StéphaneGamsjaeger, SonjaSavatier, PierreGandasi, Nikhil R.Savica, RodolfoGandhi, Jatin SundershamSavio, MonicaGangadaran, PrakashSawicka, EmiliaGantenbein, BenjaminSaxena, VikasGao, GarySayed, AhmedGao, PeisongSayed, Ibrahim M.Gao, ShouguoSazonova, Margarita A.Gao, ShuaiScalia, RosarioGaponenko, VadimScarano, AureliaGarab, GyőzőScarpa, FabioGaragiola, UmbertoScarpelli, PaoloGaragna, SilviaScarpellini, EmidioGarban, FrédéricScattolini, ValentinaGarcia Alvarez, OlgaScaturro, DalilaGarcía Gonzalo, FrancescSchäfer, KatrinGarcia Gutierrez, LuciaSchallmoser, KatharinaGarcía Meilán, IreneSchemmer, PeterGarcía Souto, DanielSchiedat, FabianGarcía, GabrielaSchierwagen, RobertGarcia, VictorSchildhaus, Hans-UlrichGarcia-Alvarez, AnaSchirhagl, RomanaGarcía-Ayllón, María-SaludSchirmer, BastianGarcía-Barrado, María JoséSchirone, LeonardoGarcia-Gil, MercedesSchlecht-Louf, GéraldineGarcía-González, VíctorSchlegel, PatrickGarcía-Guerrero, EstefaníaSchlein, ChristianGarcia-Macia, MarinaSchlereth, Simona L.García-Murria, María J.Schlüter, Klaus-DieterGarcía-Plazaola, José IgnacioSchmaier, Alvin H.García-Ríos, EstéfaniSchmetterer, KlausGarcía-Silva, SusanaSchmid, NinaGardikis, KonstantinosSchmidl, DoreenGardoni, FabrizioSchmidt, MartinaGarg, ManojSchmidtke, GunterGarmash, Elena V.Schmitz, ThomasGarofalo, StefanoSchneider, KeithGarozzo, DomenicoSchneider-Maunoury, SylvieGarstka, Malgorzata AnnaSchneitz, KayGarten, AntjeScholte, FlorineGarvin, DavidSchoot, Reineke A.Gasbarrini, AlessandroSchreml, StephanGasiūnas, GiedriusSchrock, MorganGasparini, CleliaSchröder, AgnesGasperi, ValeriaSchröder, Henrik DaaGassaway, Brandon MarkSchroeder, AnnaGaßler, NikolausSchrottmaier, Waltraud C.Gastaminza, GabrielSchryvers, AnthonyGaston, J. S. HillSchubert, Frank RichardGates, ColinSchubert, UlrichGatzoulis, Konstantinos A.Schueler, JuliaGaudio, EugenioSchuler, HerwigGauthier, BenoitSchulze, HaraldGautier, MathieuSchulze-Hentrich, JuliaGavaldà-Navarro, AleixSchulze-Osthoff, KlausGee, Christine E.Schulze-Tanzil, Gundula GesineGęgotek, AgnieszkaSchumacher, AnneGehrke, ThomasSchuurmans, CarolGeisinger, AdrianaSchuyler, Scott C.Geismann, ClaudiaSchwarzer, RolandGenc, SerminSchwenkenbecher, PhilippGenders, AmandaSciandra, FrancescaGenikhovich, GrigoryScicchitano, StefaniaGentile, AlessandraScimone, ConcettaGentile, AntoniettaScorpio, DianaGentile, FabrizioScupoli, Maria TeresaGentile, VittorioSebastian, RobinGeoffroy, Cédric G.Šeda, OndřejGeorgakilas, AlexandrosSedgewick, JerryGeorge, RajaniSedykh, SergeyGeorges, StevenSeethala, Raja R.Georgescu, AdrianaSegatto, MarcoGerard, LudovicSeger, RonyGerasimenko, OlegSegu, MarziaGerbal-Chaloin, SabineSegundo, Fayna Diaz-SanGerbeau-Pissot, PatriciaSehgal, DeepmalaGerhold, KerstinSeibler, PhilipGeri, PietroSeifalian, AlexanderGerlich, WolframSeiler, Magdalene J.Germana, AntoninoSeiliez, IbanGerrard, GarethSellal, Jean-MarcGersch, MalteSelzer, Michael E.Gershon, MichaelSemczuk, AndrzejGertsch, JürgSemenyuk, PavelGessi, StefaniaSempere, LorenzoGeuna, MassimoSen, Monokesh KumerGeva-Zatorsky, NaamaSenetta, RebeccaGhanim, BahilSenis, YotisGhasemi, MehdiSeo, Jae-HongGhasghaei, TroySeracchioli, RenatoGherardi, GaiaSeralini, Gilles-EricGhezzi, DanieleSerbanovic-Canic, JovanaGhiasi, Seyed MojtabaSereno, DenisGhimire, KedarSereti, EvangeliaGholamreza, GohariSerge, PlazaGiacomini, AriannaSergeeva, OlgaGianesello, LisaSeroogy, Kim B.Giangrande, AngelaSerra, AidaGiangrieco, IvanaSerra, Maria PinaGiannoccaro, Maria PiaSerrador Peiró, Juan M.Gibeaux, RomainSerra-Moreno, RuthGibellini, LaraSerrano, EmmanuelGiblin, FrankSerrano-Mollar, AnnaGibson, SpencerSerrano-Pozo, AlbertoGica, NicolaeSerratì, SimonaGieras, AnnaSesillo, Francesca BoscoloGigant, BenoîtSessa, FrancescoGigante, AntoniettaSestito, RosannaGijbels, EvaSethi, GautamGil Fernández, VanessaSevastre, BogdanGil, Cristiane DamasŠevčík, JanGil, ZivSeverino, PaoloGillespie, Peter G.Sfikakis, Petros P.Gillet, ReynaldSgarbanti, MarcoGimble, JeffreyShah, VaibhavGioia, MagdaShahbaaz, MohdGiovambattista, AndrésShahbazi, Marta N.Giovarelli, MatteoShakibaei, MehdiGiovarelli, MirellaShalaby, SarahGiraud, Marie NoëlleShanely, R. AndrewGirault, AlbanShao, Wen HaiGires, OlivierSharakhov, IgorGiri, Anil K.Sharif, JafarGirolamo, FrancescoSharma, DipaliGirstun, AgnieszkaSharma, JaiprakashGissen, PaulSharma, TriptiGiuffrida, RosarioSharoar, Md. GolamGiurisato, EmanueleShashikanth, NiteshGiuseppina, BasiniShaul, Yoav D.Gkogkas, ChristosShaul, YosefGkretsi, VasilikiShaykhiev, RenatGladish, Daniel K.Shearer, CameronGlaser, KarinShekhova, ElenaGlover, DavidShen, ChangHuiGnanapragassam, Vinayaga SrinivasanShen, HaihongGnanasundram, Sivakumar VadivelShepherd, TrevorGobeil, Fernand, Jr.Sheth, BhavwantiGoda, NobuhitoShevach, EthanGoel, Peeyush N.Shi, De-LiGoh, Sung-HoShi, HaoshenGokhale, Nandan S.Shi, JiajunGoldstein, AllanShi, JianGołembiowska, KrystynaShi, Patricia A.Golenhofen, NikolaShiba, KiyotakaGolia, EvangeliaShibata, YasushiGolovics, Petra AnnaShie, Ming-YouGomarasca, MartaShigdar, SarahGomes, Ana RitaShih, Yin-HwaGomes, Eduardo D.Shiina, NobuyukiGomes, FabioShim, Jae-HyuckGomes, Joao R.Shim, Joon W.Gómez Orte, Eva MaríaShimada, YohtaGomez, NidiaShimakawa, GingaGómez-Torres, Maria JoséShimaoka, MotomuGomzikova, MarinaShimizu, TakatsuneGonçalves, AnaShimizu, TaroGonçalves, Céline S.Shimokawa, NaofumiGonfiotti, AlessandroShin, Crystal S.Gonkowski, SławomirShin, KichulGonzalez Espinosa, ClaudiaShinozaki, YoshihitoGonzalez Torralva, FidelShirahata, TatsuyaGonzalez, AntonioShirakawa, HitoshiGonzalez, Francisco GarciaShizu, RyotaGonzalez, VictorShortland, PeterGonzález-Arenas, AlieshaShu, XinhuaGonzález-Espinosa, ClaudiaShukla, KirtikarGonzález-Fernández, CarlosShvetsova, Anastasia A.Gonzalez-Menendez, PedroSibille, NathalieGonzález-Mesa, ErnestoSierra-Campos, ErickGonzález-Navarro, HerminiaSiewert, KatherinaGonzalez-Pujana, AinhoaSilachev, DenisGonzález-Reyes, AcaimoSilva, DianaGopal, KeshavSilva, Nuno A.Gorbacheva, LiubovSilva, Severino Jefferson RibeiroGorbatyuk, Oleg S.Silver, Frederick H.Gorbe, AnikoSilvestre, Miguel AngelGorchakov, Andrey A.Silvestre-Roig, CarlosGorgulu, KivancSimić, TatjanaGorio, AlfredoSimoes, Davina Camargo MadeiraGórna, Maria W.Simon, Hans-uweGorrie, Catherine AnneSimone, JonathanGorvel, LaurentSimos, GeorgeGoswami, MukundaSimpson, Julie E.Goto, YuheiSince, MarcGotta, MonicaSinenko, Sergey A.Götz, Klaus-PeterSingh, KarmveerGoudarzi, SalmanSingh, Kasha PriyaGould, RobertSingh, Meenesh R.Gozal, EvelyneSingh, NishaGrabarek, Beniamin OskarSingh, Shashi ShekharGrabowska, IwonaSingh, Sitanshu S.Gracia Lostao, Ana IsabelSingh, Zeba NiaziGrade, SofiaSini, Maria CristinaGraef, MartinSinigaglia, AlessandroGräf, RalphSinigaglia, ChiaraGragnano, FeliceŠinko, GoranGraham, AnnetteSioofy-Khojine, AmirbabakGraille, MarcSioud, MouldyGramignoli, RobertoSipeki, NóraGramolelli, SilviaSipos, KatalinGranados-Principal, SergioSirico, DomenicoGranados-Principal, Sergio M.Sirois, Martin G.Granito, AlessandroSiska, Peter J.Granot, ZvikaSisto, FrancescaGrãos, MárioSitzia, ClementinaGrassi, GuidoSivakumar, SushamaGrau, James W.Skinner, PamelaGraumann, PeterSkladany, LubomirGray, Douglas A.Skok, MarynaGraziella, MessinaSkonieczna-Żydecka, KarolinaGreber, UrsSkuli, NicolasGreene, Nicholas P.Skup, MalgorzataGreenhalgh, DavidSkuratovskaia, DariaGrefhorst, AldoSkuza, LidiaGregor, MartinSlaaby, RitaGregory, Christopher D.Slade, NedaGreiner, JohannesSławińska, UrszulaGrela, PrzemysławSleigh, JamesGreotti, ElisaSlijepcevic, PredragGrespan, EleonoraŚliwiński, TomaszGrewal, ThomasSlominski, AndrzejGrieve, DavidSlovin, SusanGrigorov, IlijanaSmedowski, AdrianGrigoryan, Eleonora N.Smeland, SigbjørnGrillo, ElisabettaŚmigiel, RobertGrimm, MarcusSmith, Gary D.Grimm, W. D.Smythe, ElizabethGristina, RobertoSo, Jae-SeonGrobe, KaySoares, SylviaGröbner, GerhardSoba, PeterGrocholewicz, KatarzynaSobinoff, AlexanderGroschner, KlausSobrido-Cameán, DanielGrossman, Lawrence I.Sodeik, BeateGrote, KarstenSofinska, KamilaGrove, JoeSohrabi, YahyaGroveman, BradleySoilleux, ElizabethGruba, NataliaSoldan, Samantha S.Grusch, MichaelSoldati, GianniGrützkau, AndreasSoler, CarlesGryder, BerkleySolomonov, InnaGrzela, KatarzynaSolovchenko, AlexeiGrzmil, PawełSommer, Grün NicoleGrzymajlo, KrzysztofSong, Dong-KeunGrzywa, TomaszSong, JaewhanGu, SiyiSong, Jun-HoGu, WeiSong, RuiGu, ZhenglinSong, WenxiaGualberto, JoseSong, ZiyiGuang, ShouhongSoni, ChetnaGuatimosim, SilviaSonntag, KaiGude, Natalie A.Sontake, VishwarajGudermann, T.Soontorngun, NitnipaGueron, GeraldineSordi, ValeriaGuerra, FloraSorelli, MarcelaGuerrero, AdanSoreq, HermonaGuerrero, Sandra M. MartinSorg, OlivierGuescini, MicheleSoria, Federico N.Guevara Garcia, Angel ArturoSoriano-Romaní, LauraGuevara González, RamónSoriano-Sarabia, NataliaGuha, PrasunSorice, MaurizioGuihot, AmélieSorio, C.Guillamat-Prats, RaquelSorokin, Vitaly A.Guillén Viejo, CarlosSorokina, OksanaGuillén, Carlos ViejoSorrentino, RaffaellaGuillen-Grima, FranciscoSorrentino, VincenzoGuilpain, PhilippeSortino, Maria AngelaGuimarães, FernandoSoshnikova, NataliaGuimarães, Simone E. F.Sosic, IzidorGuinamard, RomainSossin, WayneGuiot, CaterinaSoulika, AthenaGuix, Francesc XavierSoundararajan, MeeraGüldiken, NurdanSousa, Mónica MendesGulino, RosarioSouyri, MicheleGulluni, FedericoSpagnuolo, CarmelaGulya, K.Spampinato, SantiGulyaeva, Natalia V.Sparagna, Genevieve C.Gumerov, Vadim M.Sparrer, Konstantin M. J.Guo, LilongSpartalis, Michael D.Guo, QingbinSpeake, CateGuo, YongSpears, NorahGupta, AnishaSpencer, Peter S.Gupta, KuldeepSperdouli, IlektraGurevich, Vsevolod V.Spike, Benjamin T.Gurskaya, Nadya G.Spinelli, LauraGurzov, Esteban N.Spite, MatthewGutiérrez, LauraSpletter, MariaGutiérrez-Cañas, IreneSposi, Nadia MariaGutierrez-Juarez, RogerSquatrito, MassimoGutmann, MarcusŠrámek, JanGuttery, David S.Srivastava, AkritiGuzhova, Irina V.Sriwastva, Mukesh KumarGyoergy, BenceStaal, Frank J. T.Hadjipavlou-Litina, DimitraStacchiotti, AlessandraHagedorn, MonicaStahl, MirjamHahne, JensStančiaková, LuciaHaigh, AnthonyStandal, ThereseHajduch, EricStanescu-Spinu, Iulia-IoanaHakovirta, Harri H.Stanislawska-Sachadyn, AnnaHale, Taben M.Stanke, FraukeHalle, StephanStark, Lesley A.Haller, ThomasSt-Arnaud, ReneHalliwell, Robert F.Starobova, HanaHalloran, KieranStarzyński, RafałHálová, IvanaStathopulos, PeterHam, DanielStauber, TobiasHamad, Mawieh A.Staudinger, Jeff L.Hamaguchi, TsuyoshiStayner, CherieHamaratoǧlu, FisunStecco, CarlaHamazaki, JunSteenbrugge, JonasHamieh, MohamadStefan, SimmHamilton, RussellSteffen, ImkeHammer, Sandra BeerŠtefulj, JasminkaHammers, Christoph M.Steinman, GaryHamon, MorganStellos, KonstantinosHamperl, StephanStenmark, KurtHan, InboStepanov, Alexey V.Han, JaeseokStephens, Jacqueline M.Han, PingpingStępniewski, JacekHan, RenzhiStevens, Claire H.Handa, Avtar K.Stevenson-Lerner, HeatherHanga, Mariana Petronela (Petra)Stewart, JasonHannan, Katherine M.Stewart, SarahHanschen, Erik R.Stewart-Ornstein, JacobHappel, ChristineStimpfel, MartinHaraguchi, TokukoStinghen, Andréa Emilia MarquesHardien, DavidStival, CintiaHardtke-Wolenski, MatthiasStocco, CarlosHardy, Rowan S.Stokin, Gorzard BernardHarel, AmnonStorchi, LorianoHargreaves, IainStott, Jennifer B.Hargreaves, Iain ParrySt-Pierre, Marie V.Harijith, AnanthaStrafella, ClaudiaHärkönen, PirkkoStratmann, BerndHarlé, AlexandreStratton, RichardHarper, Matthew T.Straube, JasminHarris, David T.Strazhesko, IrinaHarris, Norman R.Stricker, SigmarHarris, RaymondStrosznajder, Joanna B.Harrison, PatrickŠtrumfa, IlzeHartley, OliverStrzelecka-Kiliszek, AgnieszkaHartman, MariuszStuart, David T.Hartmann-Petersen, RasmusStukenborg, Jan-BerndHaruna, TakenoriSu, Chih-ChiaHascall, VincentSu, QiaozhuHasegawa, NatsukiSubhi, YousifHashimoto, MakotoSubramanian, SowmyalakshmiHasipek, MetisSudol, MariusHassan, Sherif T. S.Sueyoshi, TatsuyaHatem, Stéphane N.Sugawara, AkiraHatori, YutaSugawara, ShigekiHatzimichael, EleftheriaSugaya, MakotoHatzoglou, MariaSugii, HidekiHau, PeterSugimoto, Michelle A.Hau, Raymond K.Suhito, Intan RosalinaHaugsten, EllenSul, Jai-YoonHauptmann, SteffenSuleiman, M.-SaadehHautz, TheresaSuliman, Hagir B.Hawinkels, LukasSullivan, ConHayashida, KenjiSullivan, Patrick G.Haybaeck, JohannesSun, Alfred XuyangHe, BaoyeSun, XiaodongHe, JunminSun, XingminHe, LiangcanSun, YeHe, LingSun, YinHe, XiaochenSunter, Jack D.He, YaowuSurapaneni, Krishna MohanHeath, PaulSurdo, Nicoletta ConcettaHebeda, Cristina B.Surman, MagdalenaHecker, AndreasSurugiu, VictorHedman, KlausSuryadevara, VidyaniHedwig, Hedwig Sutterlüty-FallSutanto, HenryHegde, VijaySutherland, James D.Hegermann, JanSutter, AndreasHeidor, RenatoSuzuki, AussieHeidstra, RenzeSuzuki, KazuhitoHeijman, JordiSuzuki, SusumuHeinisch, JürgenSuzuki, Yuichiro J.Heinrich, BerndSvalbe, BaibaHeinz, William F.Svensson, Mattias N. D.Hejmej, AnnaSvirskis, SimonsHelgason, VignirŚwiątkiewicz, IwonaHellemons, Merel E.Swiech, KamillaHeller, JanoschSyed, NaweedHellmen, EvaSylow, LykkeHellweg, ChristineSzablewski, LeszekHelm, AlexanderSzabo, IldikoHelmbacher, FrançoiseSzabó, RenátaHendrikx, MarcSzakiel, AnnaHendrix, MarySzanto, MagdolnaHeng, TracySzatmari, Erzsebet MariaHengst, LudgerSzczepankiewicz, AleksandraHengstler, JanSzczepańska, MariaHénique, CaroleSzczepek, Agnieszka J.Henrich, DirkSzeberény, JózsefHenriksson, JohanSzekeres, MáriaHenriques-Pons, AndreaSzeri, FlóraHenry, Linda L.Szewczyk, AdamHenry, Ryan A.Szibor, MartenHenson, JeremySzigeti, KingaHerbort, Carl P.Szoke, EvaHermouet, SylvieSzokodi, IstvánHernández, M. LuisaSzulcek, RobertHerndler-Brandstetter, DietmarSzumera-Cieckiewicz, AnnaHerr, IngridSzychowski, Konrad A.Herrbach, EtienneTaanman, Jan-WillemHerrera, Antonio J.Tabarkiewicz, JacekHerrero Ezquerro, María TrinidadTabbò, FabrizioHervera, ArnauTaboada-Serrano, PatriciaHeß, JochenTaha, ZaidHessheimer, Amelia J.Tahirovic, SabinaHester, JoannaTaija, MäkinenHetman, MichalTaillandier, DanielHeumann, RolfTailleux, AnneHidalgo, JuanTakabatake, KiyofumiHilfiker, SabineTakahashi, ChiakiHilhorst, MarcTakahashi, ToshioHill, April L.Takam Kamga, PaulHill, ReginaldTakeda, ShunichiHimanshu, AroraTakeuchi, Jun K.Hime, GaryTalaverón, RocíoHimoto, TakashiTamaian, RaduHinds, Terry D.Tamarit, JordiHippler, MichaelTamba, Bogdan IonelHirakawa, YosukeTamkovich, SvetlanaHirano, KatsuyaTampa, MirceaHirata-Tsuchiya, ShizuTan, Tse-HuaHiromatsu, YujiTan, Tuan ZeaHirsch, EmilioTanaka, Hiroyoshi Y.Hitomi, KiyotakaTanaka, MiwaHizi, AmnonTanaka, NobuyukiHo, Gwo-TzerTanaka, SatoshiHochheiser, KatharinaTanaka, TakujiHochrainer, KarinTancredi, VirginiaHofer, EdithTandon, RiteshHofer, Matthias D.Tang, AnnaHoffer, BarryTang, Chih-YungHoffmann, UweTaniguchi, HiroakiHogue, Brenda G.Taniguchi, MasaakiHohenfellner, KatharinaTanner, Jessie C.Hohmann, TimTanos, BarbaraHojan, KatarzynaTantos, ÁgnesHollmén, MaijaTao, JunyanHollywood, Jennifer A.Tao, ZhipengHolubova, MonikaTao-Cheng, Jung-HwaHolzer, MaxTaouis, MohamedHombauer, HansTarallo, ValeriaHomma, TakujiroTarantino, GiovanniHonaramooz, AliTarantino, UmbertoHong, Chien-TaiTarasov, AndreiHong, Jiann-RueyTartey, SarangHong, Seok-HoTaru Sharma, GutullaHong, XupengTasaki, OsamuHoppler, StefanTaschler, UlrikeHori, ToshiyukiTasken, KjetilHorsfall, LauraTassieri, ManlioHorsfield, Julia A.Tateno, ToruHortobagyi, TiborTátrai, PéterHoshiba, TakashiTattini, MassimilianoHoshino, FumiTavian, DanielaHosui, AtsushiTawk, MarcelHo-Tin-Noé, BenoîtTaylor, Andrew W.Hottiger, MichaelTaymans, Jean-MarcHotto, Amber M.Tchoghandjian-Auphan, AurélieHough, KennethTeague, Heather L.Houhamdi, LindaTeasdale, RohanHouse, ImranTeijeiro, Juan ManuelHoutman, JonTeixeira, José H.Hovatta, IirisTeixidó, CristinaHoyer, JulianeTeneva, IvankaHoyer-Fender, SigridTeodoro, JoãoHritcu, LucianTeper, Sławomir JanHsiao, ChengchihTeraa, MartinHsiao, Hui-HuaTerao, MinekoHsieh, Ming-JuTerentyev, Vasily V.Hsin, I-LunTerlizzi, MichelaHsu, Li-ChiTerry, CassandraHsu, Li-SungTesar, VladimirHsu, Ren-JunTesarik, JanHuang, CaoTessem, Jeffery S.Huang, Chiung-KueiTesti, Anna MariaHuang, HuTestoni, BarbaraHuang, HuocongTews, BirkeHuang, JingThakur, ShilpaHuang, LeiThangavel, ChellappagounderHuang, MingchaoThankam, Finosh G.Huang, Shar Yin NaomiTheoharides, Theoharis C.Huang, ShuanglongTheopold, UlrichHuang, Shu-PinTheotokis, PaschalisHuang, ZixiaTheret, MarineHuber, Eva M.Thevenot, Paul T.Huber, MichaelThiam, Hawa RacineHuber, ReneThielemann, ChristianeHuber, SamuelThiem, AlexanderHudson, NatalieThierry, AlainHueso, MiguelThomas, CharlesHughes, TimothyThongprayoon, CharatHuh, Jin HoeThorne, PeterHukowska-Szematowicz, BeataThorsby, Per MedbøeHumphries, BrockTian, XiaoyuHung, Chung-LiehTiberio, LauraHung, Shih-YaTilocca, BrunoHuo, YuqingTimmins, JoannaHuot, Joshua R.Timofeev, SergeyHur, Jae YoungTissier, Paul LeHurley, James B.Tixeira, RochelleHusa, Ana-MariaToapanta, Franklin R.Husnjak, KoraljkaTochigi, YukiHussain, Ali A.Toczek, JakubHwang, HeeyounTodorović, AnaHwang, In KooTodorovic, SlobodanHwang, InhwanTodt, DanielHytti, MariaToffan, AnnaIaccarino, CiroTogashi, MarieIaccino, EnricoTogashi, TatsuyaIacoangeli, AlfredoToh, Wei SeongIakimova, Elena T.Toietta, GabrieleIannelli, DomenicoTokarek, TomaszIbrahim, Sulaiman S.Tokunaga, FuminoriIchii, HirohitoTolkacheva, Alena G.Ientile, RiccardoTolwinski, NicholasIevinsh, GederstTomar, DhanendraIevinsh, GedertsTomasello, Marianna FloraIguchi, YoheiTomaszewska, EwaIijima, NoriakiTomov, Martin L.Ijima, HiroyukiTomuleasa, CiprianIkeda, MasahiroTonazzini, IlariaIkegami, TadashiTonelli, MicheleIlchibaeva, TatianaTong, QingyiIlle, FabianTong, XinImai, YasuoToporowska, MagdalenaImamura, MasahiroTorgasheva, AnnaImbach, Paul A.Törnquist, KidImbert, VéroniqueTorras, JuanImmenschuh, StephanTorres, RaulImperio, DanielaTorsello, AntonioInes, LuisToscani, DeniseInfante, ArantzaToshimitsu, YamaokaInga, AlbertoTossetta, GiovanniIngley, EvanTosti, ElisabettaIniesta Cuerda, MaríaToth, RachelInoue, TakafumiTotolian, AregInyushin, Mikhail Y.Totta, PierangelaIommelli, FrancescaTouil-Boukoffa, ChafiaIop, LauraToyoda, MasashiIorio, EgidioTraiffort, ÉlisabethIqbal, JahangirTraina, GiovannaIrfan, MohammadTraini, ToninoIrini, EvnouchidouTraka, MariaIsaeva, Marina P.Tramutola, AntonellaIshigaki, YasuhitoTran, Hai BacIshihara, KatsuhikoTranbarger, Timothy JohnIshikawa, KiyotakeTranquilli Leali, Paolo TranquilliIshikawa, YasuyukiTranter, MichaelIshino, YoshizumiTrapero, IsabelIskender, IlkerTreglia, GiorgioIslas Osuna, Maria AuxiliadoraTrendelenburg, MartenIsupov, MichailTrentini, AlessandroItatani, YoshiroTribouillard-Tanvier, DéborahIto, GentaTribulova, NarcisIto, HisashiTricarico, DomenicoIvan Jose, VechettiTrifilieff, PierreIvanova, MargaritaTriggiani, V.Ivković, VanjaTrincavelli, Maria LetiziaIwaszkiewicz-Grzes, DorotaTringali, StephaneIyer, AishwaryaTrinkaus-Randall, VickeryIyoda, MasayukiTripathi, AshutoshIzawa, TakashiTroadec, Jean-DenisIzdebska, MagdalenaTroadec, Marie-BérengèreJablonska, EwaTroncoso-Ponce, AdrianJabłońska, JadwigaTrouche, DidierJackowiak, HannaTrouillas, JacquelineJackson, Joshua G.Trounson, AlanJackson-Weaver, OlanTruchado, MartaJacob, FadiTrzcińska, MonikaJacobo-Herrera, Nadia JudithTsagareli, Merab G.Jacobsen, ElizabethTsagozis, PanagiotisJacquelot, NicolasTsai, Kun-LinJacquemet, GuillaumeTsai, RongkungJacquot, YvesTsaniklidis, GeorgiosJadiya, PoojaTsikou, DanielaJafari, AbbasTsimokha, Anna S.Jafri, MohsinTsou, Wei LingJaganjac, MoranaTsuchiya, HiroyukiJaime, Diego FrancoTsuge, TomohikoJain, ManishTsukamoto, ShoJakimowicz, DagmaraTsukuba, TakayukiJakovlevic, DraganaTůma, ZdeněkJalkanen, SirpaTungjai, MontreeJames, JoelTura-Ceide, OlgaJames, Richard G.Turu, GáborJamroz-Wisniewska, AnnaTuttolomondo, AntoninoJancura, DanielTutusaus, AnnaJanda, ElzbietaTuyaerts, SandraJang, HyosunTvrda, EvaJang, KyuYunTvrdá, EvaJang, Sung-WukTwumasi Osei, EmmanuelJankowska, ElzbietaTyler, JessicaJansen, ChristianTylzanowski, PrzemkoJansen, DiahannTyrkalska, SylwiaJansen, GertTyukmaeva, Venera I.Jansson, DeidreTzanoudaki, MariannaJaraiz, MyriamTzen, Jason T. C.Jarome, TimothyTzoneva, RumianaJarret, RobertUchil, Pradeep D.Jarząb, BarbaraUddin, Md JamalJasek, MonikaUeda, JunJastrzebska, BeataUeno, HiroshiJavaheri, TaherehUlasov, IlyaJavier Jiménez-Jiménez, FelixUllah, GhanimJavier, Raya-González,Ullevig, SarahJayawardena, Thilina U.Ulvmar, Maria H.Jazirehi, Ali R.Um, Ji WonJégo, GaëtanUmansky, SamuilJekabsone, AisteUmbarkar, PrachiJeltsch, MichaelUmehara, MikihisaJeong, Goo-BoUmemura, NaokiJeong, Se KyooUngar, DanielJeschke, UdoUngurianu, AncaJeseta, MichalUnternaehrer, JuliJeyaseelan, SamithambyUphoff, StephanJezierski, AnnaUras, Iris Z.Jha, NarendraUrbańska, Ewa M.Jhanji, VishalUrciuolo, AnnaJia, BaoleiUroš, AndjelkovićJiang, Jhih-HangUsaj, MarkoJiang, LiUshimaru, TakashiJiang, PeihuaUsta, OsmanJimbo, MitsuruUsui, TatsuyaJiménez-González, GemaUysal-Onganer, PinarJiménez-Mateos, Eva MaríaUziel, OritJochum, ChristophVaccaro, MariaJoffre, CarineVafiadaki, ElizabethJohansson, AndersVagnozzi, RonaldJohnson, DanVago, JulianaJohnson, LuiseValcke, RolandJohnstone, CameronValdés-Baizabal, CatalinaJohnstone, EdwardValdez-Jasso, DanielaJohnstone, ElizabethValenti, MariaJolicoeur, MarioValenzuela, RitaJoly, Jean StéphaneValiño-Rivas, LaraJones, MarjorieValjent, EmmanuelJorge, ErikaValkov, EugeneJorrin-Novo, Jesus V.Vallejo, AlejandroJoshi, AmitValles, Soraya L.Joshi, Pushpa RajVamanu, EmanuelJoshi-Barve, SwatiVan De Worp, Wouter R. P. H.Joubert, AnnieVan Den Broek, DaanJoubert, BastienVan Der Horst, GerhardJouhet, JulietteVan Der Kuyl, AntoinetteJovanov-Milošević, NatašaVan Der Laan, MartinJuan, ManelVan Der Vorst, EmielJuarranz, Yasminavan Dievoet, Marie-AstridJuiz-Valiña, PaulaVan Eijk, MarcoJullienne, AmandineVan Ginderachter, JoJulovi, Sohel M.van Gisbergen, Klaas P. J. M.Jung, Cho RokVan Gisbergen, Marike W.Junion, GuillaumeVan Grevenynghe, JulienJunker, AnnaVan IJzendoorn, DavidJuranek, Judyta KarolinaVan Maele, LauryeJurášek, MichalVan Nieuwenhoven, Frans A.Jurič, Damijana MojcaVan Ry, PamJurisic, VladimirVan Soom, AnnJurk, KerstinVan Vliet, Kim M.Jurkowska, Renata ZofiaVan Wuytswinkel, OlivierKabir, Mohammad FaujulVan Zonneveld, Anton JanKachamakova-Trojanowska, NeliVandekerckhove, BartKaether, ChristophVanden Heuvel, Gregory B.Kaewput, WisitVandevoorde, CharlotKahraman Dirice, SevimVandewalle, JolienKaji, HiroshiVan-Dijk, JulietteKalamajski, SebastianVandooren, JenniferKaleta, BeataVanneaux, ValérieKalirai, HelenVannini, EleonoraKalmann, RachelVannucchi, Maria-GiulianaKaltenecker, DorisVaqué, José PedroKaltschmidt, ChristianVáradi, CsabaKamara, MohamedVarani, GabrieleKamen, AmineVarga, ElisabethKamihira, MasamichiVarin, AudreyKaminska, BozenaVarju, CeciliaKanai, YutaVarna-Pannerec, MarianaKanamarlapudi, VenkateswarluVarricchio, EttoreKanaujiya, JitendraVaschetto, RosannaKanda, TatsuoVasileva, LiliyaKandasamy, PalanivelVatter, HartmutKandasamy, Richard KumaranVaucher, ElvireKandeel, MahmoudVaverka, MiroslavKaneda, ShoheiVavvas, Demetrios G.Kang, EunjuVaz, Ana RitaKang, JeehoonVaz, RaquelKang, JianmingVazquez, Elba S.Kang, Ju-HeeVecino, ElenaKang, Kyung PyoVedunova, Maria V.Kang, Young-HeeVega Alvarez, Jose AntonioKano, KiyoshiVega, Francisco M.Kapetanaki, MariaVega-Benedetti, Ana FlorenciaKapus, AndrásVelardi, EnricoKapustin, AlexanderVelasco, IvánKaragiannis, EvangelosVelayutham, MurugesanKarakesisoglou, IakowosVelazquez, LauraKarakostis, KonstantinosVelentzas, Athanassios D.Karalija, ErnaVelepic, MarkoKaramichos, DimitriosVelikova, TsvetelinaKaranika, StylianiVeltkamp, MarcelKarasawa, KenVenetucci, LuigiKarasuyama, HajimeVenkatesan, ShriramKarbowniczek, JoannaVennerstrom, JonathanKardassis, DimitrisVentura, CéliaKardos, JuliannaVercoutter-Edouart, Anne-SophieKarimi, KhalilVerdurmen, Wouter P. R.Karisola, PiiaVergadi, EleniKarki, PratapVergara, DanieleKarlin, Eric F.Vermiglio, GiovannaKarr, TimothyVerna, RobertoKarsak, MelihaVeroux, MassimilianoKarthik, MallikaramanVerrecchia, FranckKarwowski, BoleslawVerri, TizianoKasatkina, Ludmila A.Verrier, Eloi R.Kaschina, ElenaVersino, ElisabettaKashanchi, FatahVerticchio, AliceKasimatis, Katja R.Vetter, MarcusKasirer-Friede, AnaVeyron-Churlet, RomainKasparov, SergeyVicari, Marcelo RicardoKasprzak, AldonaVicario-Abejón, CarlosKassan, ModarVicaș, Laura GrațielaKassis, IbrahimVicente, Cristina P.Kasuga, KensakuVicente, Giner GalvánKataoka, NaoyukiViczian, AndrasKataoka, Tatsuki R.Vida, CarmenKathare, Praveen KumarVidal, ReneKato, TakafumiVidal-Sanz, ManuelKatsinelos, TaxiarchisVieyra, AdalbertoKatzin, Alejandro M.Viggiano, EmanuelaKaufman, DanVijayaanand, Arokia MariyadossKaulich, ManuelVijayakumar, SarathKaur, GagandeepVilaboa, NuriaKaushik, GarimaVillareal, MyraKavanagh, DeanVillas-Boas, Gustavo R.Kawabata, AtsufumiVincent, C. TheresaKawabata, ShinjiVincent, DelphineKawaguchi, NanakoVincentini, OlimpiaKawai, TatsuoVinci, CristinaKawakami, ToshiVincze, EvaKazui, ToshinobuVirag, LaszloKazunori, TomitaVisentin, SergioKe, Liang-YinVishnoi, KanchanKekalainen, EliisaVishweswaraiah, SangeethaKeklikoglou, IoannaVisioli, FrancescoKeller, Jonathan R.Vitetta, LuisKemmerling, UlrikeVitorino, RuiKempaiah, PrakashaVives, JoaquimKempisty, BartoszVizoso-Vázquez, AngelKendall, TimVlachonasios, KonstantinosKerdine-Römer, SaadiaVlassi, MetaxiaKeresztes, AttilaVodenkova, SonaKerr, Nadine A.Vodickova, LudmilaKeshari, SunitaVoidarou, ChrissoulaKetteler, RobinVolkmer, HansjürgenKhairnar, Vishal S.Volonte, CinziaKhalil, Ahmed Mohamed AliVolta, FrancescoKhan, IlyasVon Appen, AlexanderKhan, SabbirVon Hörsten, StephanKhan, Zeeshan AhmadVon Kopylow, KathreinKhanam, ArshiVon Strandmann, Elke PoggeKhaperskyy, Denys A.Vontas, John G.Kharaghani, DavoodVorselaars, Adriane D. M.Khasabov, Sergey G.Vos, MelissaKhatami, MahinVrachatis, Dimitrios A.Kherraf, Zine-EddineVrzal, RadimKhiati, SalimVuillefroy De Silly, RomainKhodzhaeva, Zul’Fiya SagdullaevnaVuilleumier, NicolasKholová, IvanaVukicevic, SlobodanKhoo, Kay-HooiVukojevic, KatarinaKhosla, AashimaVulpis, ElisabettaKiang, Juliann G.Vydra, NataliaKiecker, ClemensWachten, DagmarKiela, PawelWade, Rachael M.Kiesel, BarbaraWadham, CarolKil, Eui-JoonWaghray, AvinashKillinger, Bryan AndrewWagner, Kay-DietrichKim, AlfredWahli, WalterKim, Hahn YoungWähnert, DirkKim, Hei-sungWainman, AlanKim, Ho YeonWakimoto, HiroakiKim, Ho-SeongWalczak, ClaireKim, Hyun KooWalczak-Drzewiecka, AureliaKim, Hyung-SikWalker, Douglas G.Kim, Hyun-JungWallings, Rebecca L.Kim, In-JungWalter, FruzsinaKim, JaebongWalter, SilkeKim, JaesangWalter, UlrichKim, JaesungWalton, RichardKim, JaeyoonWan, LeiKim, Jong-EunWandinger-Ness, Angela U.Kim, Jong-HyukWang, Aline Yen LingKim, Ki-YoungWang, Bingyan J.Kim, Kyeong-ManWang, ChihyangKim, KyoungtaeWang, Chrong-ReenKim, Kyung-MinWang, Chun-WeiKim, Min JungWang, Fun-InKim, Min-SooWang, GuanKim, Nam DeukWang, HsiuyingKim, Sahn-HoWang, Hui-ChunKim, Sang WooWang, Hung-JenKim, Sang-NamWang, Jaw-YuanKim, SeongjaeWang, Jian-YingKim, ShinWang, Jin'AnKim, Sun-youngWang, JinghuaKim, Tae-DonWang, JinliKim, WoojinWang, Lan-HsinKim, YoosikWang, LeiKim, Yu-JinWang, LingKimura, KiminoriWang, LizhongKimura, NobuyukiWang, Nan-kaiKino, TabitoWang, NifeiKinose, YasutoWang, PengKinoshita, HiroyukiWang, RichardKins, StefanWang, ShaobinKirály, LórántWang, ShuminKirches, ElmarWang, Tong-HongKireev, Igor I.Wang, XiaofengKirkland, Jacob G.Wang, XiaoxinKishi, YusukeWang, XuejunKishimoto, JiroWang, YajingKiss, AndreaWang, Yang-KaoKiss, Anna L.Wang, YigangKiss, CsongorWang, YingKiss, LorandWang, Ying-HsiuKiss, RitaWang, Yuan-HsiKiss, RobertWang, ZhaoningKissa, KarimaWang, ZhaoqiKitagawa, SeiichiWang, ZhixiangKitamura, DaisukeWang, ZhoufeiKitamura, KenjiWang, ZongjieKitano, TakeshiWant, Muzamil YaqubKitchen, PhilipWard, W. StevenKitowska, KamilaWaszkiewicz, NapoleonKitt, Jay P.Watanabe, EiichiKittel, AgnesWatanabe, MasakatsuKlaile, EstherWatanabe, ToshioKlar, AgnesWatanabe, YasuoKlavins, LinardsWatkins, Paul A.Kleeff, JörgWawrzyniak, PaulinaKlein, StefanieWax, AdamKleinman, HyndaWculek, Stefanie K.Klementieva, OxanaWeaver, Tyler M.Klempnauer, Karl HeinzWeber, K. ScottKleuser, BurkhardWeeden, Norman F.Klewicka, ElzbietaWehrens, Xander H. T.Klimaszewski, LarsWei, JunKlimczak, AleksandraWei-Cheng, TsengKlimek-Ochab, MagdalenaWeinberger, FlorianKloc, MalgorzataWeisberg, EllenKlymiuk, MicheleWeiss, ÉtienneKnapczyk-Stwora, KatarzynaWeiss, MartinKnezevic, JelenaWeissenhorn, WinfriedKnight, JimWelner, Robert S.Knuplez, EvaWelsh, MichaelKobarg, JörgWende, WolfgangKobayashi, KentaWendt, LisaKobayashi, KoichiWerneburg, SebastianKobayashi, NobumichiWerner, HaimKobayashi, SatoruWerner, SabineKobayashi, TatsuyaWernersson, SaraKobayashi, YoshiroWernly, BernhardKobeissy, FirasWesołowska, OlgaKobierzycki, ChristopherWestmark, Cara J.Koch, Karl-WilhelmWeston, StuartKochneva, GalinaWetzel, ScottKočí, ZuzanaWever, AlyssaKoduru, Srinivas V.Wheeler, DaveKoeleman, BobbyWheeler, MichaelKoestenberger, MartinWhite, FletcherKohorn, BruceWhiteside, ElizaKoinis, FilipposWibowo, ErikKoivisto, AriWichmann, ChristianKojima, KenjiWidemann, EmilieKok, Chung HoowWidiapradja, AlexanderKolackova, IvanaWiedlocha, AntoniKolaczkowska, ElzbietaWiegers, Gerrit-JanKoldehoff, MichaelWielockx, BenKolosova, NataliyaWienkoop, StefanieKołton, AnnaWigle, JeffreyKomleva, Yulia K.Wijnholds, JanKondo, TomoWikonkál, NorbertKoninckx, Philippe R.Wikström, MårtenKonjar, ŠpelaWilber, Andrew C.Konjevoda, PaškoWilhelm, JochenKonstantinidis, Theocharis G.Wille, HolgerKontaraki, Joanna E.Willerth, StephanieKonturek, Peter ChristopherWilliam Richardson, WilliamKoos, BjörnWilliams, DavidKopjar, MirelaWillis, Dianna E.Kopp, Benjamin T.Wilm, MatthiasKorać, PetraWing, SimonKorczeniewska, OlgaWinsz-Szczotka, KatarzynaKordek, AgnieszkaWinter, JochenKordowitzki, PawełWiszniewska, AlinaKordyum, Elizabeth L.Witalisz-Siepracka, AgnieszkaKorfitis, ChrysovalantisWitarski, WojciechKorkmaz, YükselWitherden, DeborahKorkotian, EduardWitkiewicz, WojciechKorolenko, Tatiana A.Włodarczyk, MarcinKorolkova, Olga Y.Włoga, DorotaKorosoglou, GrigoriosWnuk, AgnieszkaKorthagen, Nicoline M.Wolf, IdoKortholt, ArjanWolff, DanielKorzhevskii, DmitriiWołkow, PawełKosan, ChristianWon, Seok JoonKoscielny, SvenWong, Boon-SengKosowska, AgnieszkaWong, Chi-MingKostallari, EnisWong, Chun KwokKostas, MichalWong, ChungKostić, MarinaWong, Dennis T. L.Kostyuchenko, RomanWong, Ka-LeungKosuge, YasuhiroWong, Richard W.Kot, KarolinaWoo, JunsungKothari, Vishal M.Woo, Se JoonKottilil, ShyamWoo, So-YounKottmann, Andreas H. H.Wood, Thomas R.Kounatidis, IliasWoodgett, Jim R.Kovac, StjepanaWright, Peter T.Kováčik, AndrejWsół, AgnieszkaKovács, Árpád FerencWu, Ai-MinKovács, KrisztinaWu, Bo-SenKovács-Öller, TamásWu, Chia ShanKovalszky, IlonaWu, Chun-ChiKowalewska, ŁucjaWu, GuangyuKowalska, Maria AnnaWu, Huang-PinKozhevnikova, OyunaWu, JianjunKozieł, EdmundWu, LijunKozieł, MonikaWu, MinghuaKoziorowski, DariuszWu, Min-HuanKozlova, ElenaWu, QihuiKozlowska, EmiliaWu, Ray-changKrag, Thomas O.Wu, Shi-BeiKrapf, DarioWu, WilliamKrasikova, AllaWu, XiaoanKrasikova, Yuliya S.Wu, Yuh-LinKratz, Ewa MariaWuebbles, RyanKrause, MatthiasWyns, ChristineKrawiec, PaulinaWysokińska, AnnaKreis, Nina-NaomiXavier, StephenneKrick, StefanieXia, GuangbinKrishna, GokulXiao, LiKrishna, Suresh Babu NaiduXiao, LuKrishnakumar, DevadasXiao, YongliKrishnamoorthy, Raghu R.Xiaoyan, HuiKrishnasamy, GopinathXie, Lai-huaKristen W., CohenXie, LingxiaoKristian, TiborXie, ZhiqunKrohne, GeorgXie, ZhongqiuKron, IvanXie, ZuoxuKropp, MartinaXin, MeiKrskova, LenkaXing, D. A.Krstic, JelenaXiong, Tou-CheuKrueger, AndreasXirodimas, DmitrisKrüger, MarcusXu, JialinKruger, MarlenaXu, JinbinKruglov, AndreyXu, KuiKrzystek-Korpacka, MalgorzataXu, RenKu, Wei-ChiXu, SuowenKuang, ZhengXu, XuehuaKubala, LukášXu, YangKubota, HiroshiXu, Yan-MingKucharska-Mazur, JolantaXue, FengKucharz, EugeneXue, MinKuehnel, FlorianYagi, HiroshiKujan, OmarYaish, MahmoudKuliczkowski, KazimierzYakhine-Diop, Sokhna M.S.Kulik, AnnaYam, Gary Hin-FaiKulikov, Alexander V.Yamada, TakashiKulkarni, DhananjayaYamada, TaketoKulkarni, PriyankaYamamoto, AkihitoKullberg, SusannaYamamoto, MasatoKulus, DariuszYamamoto, NaokiKumar, AjayYamaoka, KunihiroKumar, AnoopYamaoka, ToshimitsuKumar, AnujYamasaki, TriciaKumar, AshokYamashita, AtsushiKumar, KishoreYamashita, KimihiroKumar, ManuYamashita, ToruKumar, MukeshYan, BowenKumar, Thallapuranam Krishnaswamy SureshYan, DayunKumari, DamanYan, HuiminKumari, MeenaYanagisawa, NaokoKumari, SonamYandeau-Nelson, Marna D.Kümmerer, Beate M.Yang, BoKundariya, HardikYang, ChuanbinKundu, KiranYang, Chul-SuKunkel, ThomasYang, HainingKunz, WolframYang, Hung-ChiKunze, MarkusYang, JialeKuo, HsiaohsuanYang, KunKura, BranislavYang, LiliKurakula, KondababuYang, LiuKurczyńska, EwaYang, Shun-FaKurimoto, TakujiYang, Wen-BinKuroda, HiroakiYang, XiulanKurokawa, ManabuYang, YingKurose, HitoshiYang-Hartwich, YangKurtz, AndreasYanzhuang, WangKuryk, LukaszYao, HeruiKuryłowicz, AlinaYao, HiroshiKushima, MikiYao, JunKuter, KatarzynaYao, TaoKuthati, YaswanthYaparla, AmulyaKutikhin, AntonYarygin, Konstantin N.Kuzel, Timothy M.Yasuda, TakakoKuźnik-Trocha, KorneliaYazdi, Homa PapoliKvandova, MiroslavaYe, JianKwakkel-van Erp, HannekeYen, Feng-LinKwiatkowska, KatarzynaYeo, SynKwok, Hoi-HinYerneni, Saigopalakrishna S.Kwon, ByungsukYesilkanal, Ali EkremKwon, Hyuk-JoonYévenes, GonzaloKwon, Oh SungYew, P. ReneeKwon, So HeeYi, Chun-XiaKyriakoulis, Konstantinos G.Yi, Joo MiL. Balint, BalintYlösmäki, ErkkoLa Porta, CaterinaYlostalo, Joni H.La Rosa, PiergiorgioYokota, HirokiLaboisse, Christian L.Yong, JeongsikLabouta, Hagar IbrahimYoo, Yeong-MinLabrador, JorgeYoon, Hyeun JoongLacal, Juan CarlosYoon, Sung-SooLach, Michał StefanYoon, YisangLacko, AndrasYoshida, KaoruLacobescu, Gabriela-EugeniaYoshimura, KotaroLacroix, BenjaminYoshino, HiideLaczmanski, LukaszYoshino, HironoriLadani, AmitYou, Hye JinLadekarl, MortenYou, Jae-SungLadilov, YuryYoum, Jae BoumLadomersky, ErikYoun, Seock-WonLagali, PamelaYoung, JessicaLahlil, RachidYssel, HansLahmer, TobiasYu, Cheng-ChiaLai, Wing-FuYu, JiaLai, YingJuYu, JiashingLallemand, FraņcoisYu, KaiLalli, EnzoYu, ShanpingLalonde, JasminYu, Wei-HsuanLam, Alan Tin-LunYu, Wen-ChungLam, YanYu, XinfangLamatsch, DunjaYu, XinzhuLambert, GillesYuan, MengLamichhane, SantoshYuemeng, JiaLamm-Shalem, NoaYumura, ShigehikoLampert, KathrinYurchenko, EkaterinaLanciotti, AngelaYutzey, KatherineLande, RobertoZaarour, Rania FaouziLandfield, Philip W.Ząbek, TomaszLang, DiZaboronok, AlexanderLangguth, PeterZacchia, MiriamLangsley, GordonZaccone, GiacomoLanzarone, GiuseppeZadmajid, VahidLapucci, AndreaZadra, GiorgiaLaskaratos, Faidon-MariosZaglia, TaniaLaskowska, MarzenaZaid, HilalLasseaux, EulalieZaizen, YoshiakiLässer, CeciliaZakłos-Szyda, MalgorzataLassot, IrénaZakrocka, IzabelaLatowski, DariuszZalewska, TeresaLautenschlaeger, TimZalewska-Janowska, AnnaLayer, LilianaZalvide, JuanLazarević, VladimirZamfir, Carmen LăcrămioaraLázaro-Diéguez, FranciscoZampieri, StefaniaLazzaro, FedericoZamyatnin, Andrey A.Le, Hoang-ThanhZandi, PeimanLebensztejn, Dariusz MarekZanella, IsabellaLebiedzinska-Arciszewska, MagdalenaZanelli, MagdaLechel, AndreZanetti-Domingues, Laura C.Lechniak, DorotaZanfirescu, AncaLeclerc, EstelleZangle, ThomasLecoq, LaurianeZapater, PedroLederkremer, Gerardo Z.Zapico, Sara CasadoLedesma, Juan CarlosZaramella, PatriziaLee, Cheok SoonZarkovic, NevenLee, Chia-HwaZarnowski, TomaszLee, Dong RyulZarobkiewicz, MichałLee, Geun-ShikŻarski, DanielLee, HankyuZatsepin, TimofeiLee, HoZatz, MayanaLee, JaeZawadzka, MalgorzataLee, Jeong-ChaeZefferino, RobertoLee, Ji HyunZelarayan, LauraLee, JuhyunZelasco, SamantaLee, Kyung-AhŻelechowska, PaulinaLee, Li-AngZemkova, HanaLee, Min YoungZemła, JoannaLee, Ming-JenZenarruzabeitia, OlatzLee, MyeongsangZeng, BaoshengLee, Sang-HanZeng, BijunLee, Seung HwanZeng, Chang-JunLee, SooyeonZeng, ShaohuaLee, StellaZentilin, LorenaLee, Sue C.Zerges, WilliamLee, Sung HoonZhang, ChunLee, Sung SikZhang, DongyunLee, Sung-JaeZhang, DuoLee, SunjaeZhang, HongquanLee, TaesicZhang, JinLee, Wai-LengZhang, NuLee, WanZhang, QiuyangLee, Wen-ChinZhang, WenjunLee, Yun-SilZhang, XiaonanLee, ZhenghongZhang, XingminLee-Sarwar, Kathleen AnneZhang, Yin HuaLefevre, Sophie D.Zhang, YuanLefrançois, StéphaneZhang, YuminLegembre, PatrickZhang, ZengdiLegendre, FlorenceZhao, LinlinLegewie, StefanZhao, XiandaLegrand, FannyZhao, ZhenjunLegrand, SylvainZheng, GuopingLehmann, Helge ImmoZheng, XiaomingLehotsky, JanZheng, Yun-WenLei, WeiZhong, QuanLeinonen, VilleZhong, WeiLeis, Jonathan P.Zhong, ZhengLeitch, HarryZhou, BishengLemcke, HeickoZhou, GuangqianLemieux, HélèneZhou, JingsongLemonnier, LoicZhou, LinliLennikov, AntonZhou, MeijuanLenzi, PaolaZhou, QingyuLeo, DamianaZhou, ShuanhuLeon Serrano, JavierZhou, XiaofengLeon, JuanZhou, ZhidongLeonardo, CostantinoZhou, ZhifangLeondaritis, GeorgeZhu, BangfuLeoni, ValerioZhu, DashuaiLeppik, LiudmilaZhu, JoeLerman, LilachZhu, MingzhaoLerner, UlfZhu, QianzhengLeskovac, Andreja R.Zhu, WeiLeszczuk, AgataZhu, WuqiangLeu, Jiann-HorngZhuang, ShougangLeung, Anthony K. L.Zhuang, XiaohongLeung, George P. H.Zhuo, ChunliuLevin, LeonardŽiarovská, JanaLevin, Lonny R.Zidar, NinaLevitsky, DmitriiZiegler, TilmanLewalle, PhilippeZieliński, MaciejLewicki, SławomirZiemssen, FockeLewis, Tylor R.Zikos, PanagiotisLezot, FredericZíková, MartinaLi Puma, Domenica DonatellaZimmer, JacquesLi, BoZimmer, SebastianLi, Chien-FengZimmer-Bensch, GeraldineLi, FengzhiZiolkowski, WieslawLi, GuangzhaoZissel, GernotLi, Jiao JiaoZivcak, MarekLi, JunŻołądek, TeresaLi, Ka WanZöller, MargotLi, LiZondler, LisaLi, Po-HsienZong, Wei-XingLi, ShangZórad, StefanLi, Shengwen CalvinZordoky, BeshayLi, TianhuZorec, RobertLi, WeiZornitsa Ivanova, KaterovaLi, WenboZsigmond, LauraLi, YafengZubareva, Ekaterina VLi, YanZubiaur, M.Li, Yi Shuan JulieZundler, SebastianLi, YutianZuo, LiLi, ZiruŽupan, IvanLiakopoulos, DimitrisZupan, JanjaLiampas, IoannisZwaans, Bernadette M. M.Lian, QizhouZwart, Wilbert


